# Forecasting suicide rates in India: An empirical exposition

**DOI:** 10.1371/journal.pone.0255342

**Published:** 2021-07-29

**Authors:** Prafulla Kumar Swain, Manas Ranjan Tripathy, Subhadra Priyadarshini, Subhendu Kumar Acharya

**Affiliations:** 1 Department of Statistics, Utkal University, Bhubaneswar, Odisha, India; 2 Department of Statistics, Ravenshaw University, Cuttack, Odisha, India; 3 Department of Research and Development, Kalinga Institute of Medical Science, Bhubaneswar, Odisha, India; 4 ICMR-Regional Medical Research Centre, Bhubaneswar, Odisha, India; College of Medicine and Sagore Dutta Hospital, INDIA

## Abstract

**Introduction:**

Suicide is a major social and health issue in India. Yearly statistics show a concerning increasing pattern of suicidal deaths in India which is higher in comparison to the global trend. There is limited evidence regarding historical analysis of suicide or any forecasting for suicide in India towards predicting the possible risks of death due to suicide.

**Methods:**

This paper examines the trend of suicide rate and characteristics of suicide victims in India, based on the longitudinal time series data over the last 50 years—collected from the National Crime Record Bureau Reports (1969 to 2018) of the Government of India. In our analysis, we have used the time series model to forecast the suicide rates in India for the next decade. ARIMA (4,1,0) model is found to be the best fit model for forecasting the data.

**Findings:**

There has been an observable and rising trend of suicide rates in India over the last five decades. The forecast indicates a continuance of rising suicide cases for an upcoming couple of years in India with a limited decline in the following years. The prediction model indicates a future relatively consistent pattern of suicide in India which does not seem to be a very encouraging trend. As we have not included the period staring the year 2020 onwards affected by Covid-19 and which has several disruptions in personal and family spaces, the projected suicide trend during the period of next two to three years (2020–22) may rise far high and then it may show a declining path. Along with this, there is a shift in means of suicide in the last couple of decades. Constituting the second-highest number of cases, Illness associated suicide was visibly a serious concern.

**Conclusion:**

The present analysis finds that there is no visible substantial relief for suicide deaths during the coming years in India. On the other hand, more extensive exploration of sample cases may provide important information for suicide prevention. Availability of detailed and more inclusive data will be highly useful for analysis and suicide preventive policies. Investment in public health care and other welfare activities like education and employment generation will yield visible positive results in suicide control.

## Introduction

Suicide is a major mental health problem that may occur at any stage within a life span of an individual. Suicide is premature mortality where a person deliberately terminates his/her own life. The effects of the suicide rate on society may be more serious because the monetary figure doesn’t include intangible costs, such as the development of psychological burden and secondary mental diseases in the bereaved [1]. There are several risk factors associated with it like stress, depression, failure in examination, the relationship breaks up, alcohol use disorder, financial problems, chronic pain, etc [2–13]. For nearly a year, the world has been suffering from the pandemic of the coronavirus. In this process, many have lost their close relatives, jobs, income, forced to leave from the place of work to place of origin, at resident or shelter, got depressed and some of them committed suicide [14–16]. In this scenario, India is the worst sufferer of Covid-19, and reporting one of the highest mental health burdens in the world draws attention around its annual suicide risk. In this context, the trends and number of suicide cases will be a significant public health indicator and defining factor from the Sustainable Development Goal (SDG) perspectives [17] for India while the country is striving to revive its economic growth in the Post-Covid-19 period.

According to World Health Organization, suicide is globally the second leading cause of death among the young population (aged 15–29 years) irrespective of gender after road accidents [18]. Moreover, it is the second leading cause of death among teenage girls (aged 15–19 years) after maternal conditions and the third leading cause of death among teenage boys (aged 15–19 years) after road accidents and violence [19]. Worldwide, there are nearly 8 lakh suicide cases registered every year. Most of the suicides (nearly 79%) occur in low- and medium-income countries [18]. Less than 1/4^th^ of suicidal cases occur in high-income countries altogether. Despite the remarkable progress of human societies in the 21^st^ century, a single person every 40 seconds still dies due to suicide [18]. On the other hand, in the context of India, a major developing country, the rate of suicide as per the National Crime Research Bureau (NCRB) grew at an 8% rate per year during the period-1997-2006 while the population of the country grew at 19% during that time [20].

Suicidal behaviors in India become one of the foremost issues where its incidence manifests a rising trend day by day [21]. Such a situation forces the policy planners and researchers to explore various socio-psychological and economic causes of suicides at the Individual as well as community or society level.

Several studies in India have been conducted on various aspects of suicide, for example, the link between suicide and its economic determinants [21,22], the effect of unemployment on suicide rate [23], the relationship between suicide rate and human development index [24,25], etc. As it is very essential to reduce the suicide rate, researchers have been suggesting some preventive measures to reduce the suicide rate in India [25], for which we must have some idea about the complexity of its growth rate.

This paper is novel in the way that it examines the trend and determinants of suicide rates in India over the last 51 years period (from 1969 to 2018) and forecasts the suicide rate for the next 10 years by using appropriate models. More so, we have attempted to study the age and sex-specific suicide rates as well as modes of suicide in India’s diverse socio-cultural space under the assumption that the study will be useful for health care planning and policies for suicide prevention. Hence, this study not only examines the determinants and spatial patterns of suicidal deaths but also examines trends of suicide rates over more than three decades to identify the methods of lowering the suicide rate in India.

## Materials and methods

### Data source and data extraction

We collected the data from National Crime Record Bureau (NCRB), India, the nodal agency for collecting the data on suicide incidences across India. National Crime Record Bureau (NCRB) functions under the Ministry of Home Affairs, Government of India and it publishes time series data on accidental deaths and suicides on annual basis from the year 1969 onwards. For this study, we have collected data on suicide for a period of 49 years (1969–2018) from NCRB annual reports on Accidental Deaths and Suicides in India (ADSI), freely available at https://ncrb.gov.in/en/adsi-reports-of-previous-years [20]. However, it may be mentioned here that the data during the year 1969 to 1988 is available only with the total number of suicide deaths, while the sociodemographic data were included in the following years with the introduction of new and different parameters further from time to time. So, the present study used the data of the period 1969–2018 for forecasting purposes while, the detailed demographic data on various attributes of suicide such as age groups, gender, professions, etc. along with the means and causes was analyzed for the year 1989 onwards.

Data have been extracted from NCRB reports, available as PDF documents, by second and third authors (MRT & SP). The year-wise suicide data, extracted from PDF documents, was entered in a Microsoft Excel spreadsheet which is also available as a separate table (available as S1 Table). In the process of data extraction and entry, the extracted data by the second author was entered in an excel sheet which was checked by the third author for consistency and correctness and vice versa for ensuring the overall data quality; the first author (PKS) monitored the entire data extraction and entry process by cross-checking. The completeness, accuracy, and consistency of the collected data were checked by the first author regularly.

### Ethical consideration

This data is available in the public domain so there is no need for ethical approval.

### Statistical analysis

Statistical analysis was executed by using *R* software version 4.0.1. The present data analyzed in the paper has three major parts i.e. firstly, it deals with the decadal trend of suicide rates according to different attributes and the second part explores with forecasting of the suicide rate for the next decade using time series models. Finally, the findings have been discussed by contextualizing various previous observations and evidence to highlight the socio-cultural implication and policy perspectives of the observed suicidal trends. Here, it may be mentioned that the time series forecasting model which is one of the major analysis approaches is one of the most widely used techniques for the prediction of future events. It rigorously studies the past observations of a time series to develop an appropriate model which describes the inherent structure of the series. One of the most popular and frequently used stochastic time series models is the Auto-Regressive Integrated Moving Average (ARIMA) model [24].

The ARIMA model has been successfully applied in the field of health as well as in different fields in the past due to its simple structure, fast applicability, and ability to explain the data set.

The ARIMA is one of the most used time series models as it takes into account changing trends, periodic changes, and random disturbances in the time series. ARIMA is suitable for all kinds of data, including trend, seasonality, and cyclicity. ARIMA modeling consists of four vital steps viz. identification of potential models, estimation of parameters in that potential models, diagnostic checking of residuals for white noise, and forecasting by taking the help of a selected model. ARIMA model is generally referred to as an ARIMA (*p*,*d*,*q*) where *p* signifies the order of autoregression, *d* denotes the degree of difference, and *q* is the order of moving average. In other words, the p and q were the number of significant lags of the autocorrelation function (ACF) and the partial autocorrelation function (PACF) plots, respectively, and d was the different order needed to remove the ordinary non-stationarity in the mean of the error terms.

ARIMA is a joint model of two models, autoregressive AR(p) and moving average MA(q), and is integrated using the difference variable d. The general formula of AR (p) and MA (q) models can be expressed in Eqs (1) and (2), respectively.

An autoregressive AR(p) model of order p can be written as:

$$y_{t}=c+\phi_{1}y_{t-1}+\phi_{2}y_{t-2}+\ldots +\phi_{p}y_{t-p}+e_{t}$$ (1)

Where c is a constant, e_t_ is a white noise *e*_*t*_ ~ *N*(0, *σ*^2^), *ϕ* = (*ϕ*_1_, *ϕ*_2_, …*ϕ*_*p*_) is the vector of model coefficients & p is a non-negative integer.

A moving average MA(q) model of order q uses past forecast errors in a regression model as

$$y_{t}=c+e_{t}-\theta_{1}e_{t-1}-\theta_{2}e_{t-2}-\ldots -\theta_{q}e_{t-q}$$ (2)

Where c is a constant, e_t_ is a white noise *e*_*t*_ ~ *N*(0, *σ*^2^), *ϕ* = (*θ*_1_, *θ*_2_, …*θ*_*q*_) is the vector of model coefficients & q is a non-negative integer.

The ARMA (p, q) process of orders p and q is defined as

$$y_{t}=c+\phi_{1}y_{t-1}+\phi_{2}y_{t-2}+\ldots +\phi_{p}y_{t-p}+e_{t}-\theta_{1}e_{t-1}-\theta_{2}e_{t-2}-\ldots -\theta_{q}e_{t-q}$$

$$\Rightarrow y_{t}=c+\sum_{i=1}^{p}\phi_{i}y_{t-i}-\sum_{j=1}^{q}\theta_{j}e_{t-j}+e_{t}$$ (3)

ARIMA (p, d, q) model can be written as:

$$y_{t}-2y_{t-1}-\ldots -y_{t-d}=c+\phi_{1}y_{t-1}+\phi_{2}y_{t-2}+\ldots +\phi_{p}y_{t-p}+e_{t}-\theta_{1}e_{t-1}-\theta_{2}e_{t-2}-\ldots -\theta_{q}e_{t-q}$$ (4)

Where, p autoregressive terms, d is the non-seasonal differences, q is the number of lagged forecast errors.

The best model is selected based on the AIC (Akaike Information Criterion), AICc, and BIC (Bayesian Information Criterion) values. A model with a minimum of these statistics is considered to be the best forecasting model. Generally, the AIC is calculated using the relation *AIC* = 2*k* − 2 log(*L*) and *BIC* = *k* * *log*(*n*) − 2 log(*L*); Where k (= p+q+1) is the number of parameters in the statistical model and L is the maximized value of the likelihood function for the estimated model.

We have used the Dickey-Fuller test for stationarity check. It is only after first order differencing stationarity was achieved and then the ARIMA model was applied. The forecast accuracy of the model was also evaluated using Mean Absolute Percentage Error (MAPE) and Root Mean Square Error (RMSE) measures. For trend analysis, chi-square for trend test was conducted to examine the association between categorical variables. Rates of suicide were calculated as cases per 1,00,000 persons. In all cases P-value <0.05 is considered statistically significant.

## Results

Table 1 shows the distribution of different attributes of suicide viz. gender, age group, education, profession, social status, means adopted and causes of suicide along with its average decadal trend over the last three decades. The chi-square test shows that the trend of suicide rate is significant over the decades for all the above-specified categories as its p<0.05.

**Table 1:** Distribution of different attributes along with the average decadal trend of suicides.

Variables	Year					P-value
	1969–1978	1979–1988	1989–1998	1999–2008	2009–2018	
Total	43038	49201	85265	114233	133103	
**Gender**						
Male			50160 (58.8)	71849 (62.9)	88180 (66.2)	<0.0001
Female			35105 (41.2)	42384 (37.1)	44923 (33.8)	
**Age Group(in years)**						
upto_14			6929 (8.1)	2815 (2.5)	4563 (3.4)	< 0.0001
15to 29			35426 (41.5)	40785 (35.7)	47376 (35.6)	
30to44			27561 (32.3)	38811 (34.0)	43969 (33.0)	
45to60			12711 (14.9)	22878 (20.0)	26293 (19.8)	
60andabove			6596 (7.7)	8944 (7.8)	10903 (8.2)	
**Education**^$^						
No Education			27276 (32.0)	26988 (23.6)	21628 (16.2)	< 0.0001
Primary(upto class–5th)			26310 (30.9)	29245 (25.6)	27590 (20.7)	
Middle(up to class–8th)			21659 (25.4)	27346 (23.9)	28765 (21.6)	
Matriculate/Secondary(up to class–10th)			14894 (17.5)	19711 (17.3)	27978 (21.0)	
Hr. Secondary/Intermediate/Pre-University (up to class–12th)			6501 (7.6)	8876 (7.8)	15598 (11.7)	
Diploma/Certificate/ITI			1001 (1.2)	1028 (0.9)	1552 (1.2)	
Graduate/degree			1669 (2.0)	2176 (1.9)	4022 (3.0)	
PG/Professionals (MBA etc.)			584 (0.7)	520 (0.5)	604 (0.5)	
Status Not Known			0	0	10300 (7.7)	
**Profession**^$^						
Housewife			19695 (23.1)	23410 (20.5)	22779 (17.1)	< 0.0001
Professionals/Salaried Persons			11055 (13.0)	13326 (11.7)	13206 (9.9)	
Students			5410 (6.3)	5690 (5.0)	8346 (6.3)	
Unemployed Persons			8263 (9.7)	9463 (8.3)	10624 (8.0)	
Self-employed Persons			34279 (40.2)	45848 (40.1)	37846 (28.4)	
Retired Persons			741 (0.9)	923 (0.8)	977 (0.7)	
Other			12703 (14.9)	15290 (13.4)	37473 (28.2)	
**Social Status**^$^						
Un-Married			23094 (27.1)	25010 (21.9)	29748 (22.3)	< 0.0001
Married			65840 (77.2)	81326 (71.2)	91864 (69.0)	
Widowed/Widower			5481 (6.4)	5198 (4.6)	3589 (2.7)	
Divorcee			1612 (1.9)	1238 (1.1)	1142 (0.9)	
Separated			3816 (4.5)	3117 (2.7)	2134 (1.6)	
Others			0	0	3852 (2.9)	
Status not known			0	0	5126 (3.9)	
**Means**						
Consuming Sleeping Pills			572 (0.7)	965 (0.8)	432 (0.3)	< 0.0001
Drowning			8565 (10.0)	8275 (7.2)	7352 (5.5)	
Fire/Self Immolation			8915 (10.5)	10509 (9.2)	9750 (7.3)	
Firearms			612 (0.7)	528 (0.5)	506 (0.4)	
Hanging			21056 (24.7)	21056 (18.4)	54149 (40.7)	
Poison			30052 (35.2)	42053 (36.8)	38617 (29.0)	
Self-inflicting Injury			572 (0.7)	454 (0.4)	638 (0.5)	
Jumping			1206 (1.4)	1895 (1.7)	2189 (1.6)	
Coming under Running vehicles			2991 (3.5)	3557 (3.1)	3894 (2.9)	
By Touching Electric Wire			632 (0.7)	861 (0.8)	832 (0.6)	
By Other Means			10093 (11.8)	11342 (9.9)	14259 (10.7)	
**Causes**						
Family Problems			12355 (14.5)	26217 (23.0)	34173 (25.7)	< 0.0001
Illness			15387 (18.0)	25161 (22.0)	24639 (18.5)	
Marriage Related Issues			2220 (2.6)	3819 (3.3)	6252 (4.7)	
Drug Abuse/Alcoholic Addiction			1012 (1.2)	1875 (1.4)	4496 (3.4)	
Love Affairs			4017 (4.7)	3563 (3.1)	4447 (3.3)	
Bankruptcy or Indebtedness			1387 (1.6)	2945 (2.6)	3438 (2.6)	
Other			49505 (58.1)	50653 (44.3)	53949 (40.5)	

^**$**^Data was available from 1995 onwards.

It can be observed that the number of male suicide victims has been increasing throughout the past three decades; whereas there is a fall in the number of suicide cases among females. In the last decade, 2009–2018 the males’ suicide victims were 66.2% nearly double as compared to female suicide victims i.e., 33.8%. Irrespective of the past decades, male suicide victims were consistently higher as compared to females.

The percentage share of suicide victims aged 15 to 29 has been the highest among all the age groups during the last 3 decades. However, the overall percentage share has been decreasing with subsequent decades, as it was 41.5% in 1989–1998, 35.7% in 1999–2008, and 35.6% in 2009–2018.

Education-wise, longitudinal trend (from 1989–1998 to 2009–2018) shows an observable division within individuals committing suicide among non-matriculates and matriculates and above groups; while the rate of suicide among non-matriculates was observed with a gradually decreasing trend during the period of 1989–1998 to 2009–2018, a significant rise in suicide among matriculates and above educated group was observed during this time (Table 1). On the other hand, the decadal trend shows the percentage share of illiterate persons was the highest (i.e., 32%) in the decade 1989–1998, the population in primary class (up to class 5^th^) was highest (i.e., 25.6%) in the decade 1999–2008 and population in the middle class (up to class 8^th^) was highest (i.e., 21.6%) in the decade 2009–2018 [Table 1]. Overall, the major proportion of the total suicide cases (>70% for the two decades between 1989 to 2008; >60% for 2009–2018) was observed to have taken place among the lower educated group (<matriculation) in the three studied decades i.e. 1989–1998, 1999–2008 and 2009–2018. Alongside, the trends of the percentage of suicide incidences decreased in all the higher educational groups above matriculation (except Higher secondary) during 1999–2008 from 1989–1998 [Table 1], while such trend took reverse pick during 2009–2018 than the previous decade in all the categories without exception; considering in numbers, cases are close to double in some of the educational groups(Hr secondary and graduate) during 2009–2018 than the previous decade. It seems the tendency of committing suicide is increasing among higher educated people while decreasing among lesser educated.

After reviewing the distribution of suicide victims by their profession for the past two decades we got that the percentage share of suicide victims has dipped for all the subcategories viz. Housewives, Professionals/Salaried persons, Students, Unemployed persons, Self-employed persons, Retired persons and Other from 1989–1999 to1999-2008. The decline in percentage share of suicide victims was observed of continuing till the last decade 2009–2018 except for students and ‘other’ categories; whereas it has gone up from 5.0% in 1999–2008 to 6.3% in 2009–2018 and 13.4% in 1999–2008 to 28.2% in 2009–2018 respectively.

Concerning the suicide data according to social status, it can be observed that, for the 20 years in recent past, the average suicide percentage among all sub-categories has decreased from 1989–1998 to 1999–2008. For the decade 2009–2018 average suicide percentages decreased in all categories except for the unmarried population, which has shown a rise of 0.4% over its previous decade. For the last decade i.e. 2009–2018, the average decadal percentage share of Un-married, Married, Widowed/Widower, Divorcee, Separated, Others and Status unknown were 22.3%, 69%, 2.7%, 0.9%, 1.6%, 2.9%, and 3.9% respectively.

Means adapted for suicide particularly for the category consuming sleeping pills, poison, jumping, and by touching electric wire have shown their increased percentage share from 0.7% in 1989–1998 to 0.8% in 1999–2008, from 35.2% in 1989–1998 to 36.8% in 1999–2008, from 1.4% in 1989–1998 to 1.7% in 1999–2008 and from 0.7% in 1989–1998 to 0.8% in 1999–2008 respectively. The Suicide rate has increased in the last two decades for the following three categories of means such as Hanging, Self-inflicting injury and by Other means from 18.4% in 1999–2008 to 40.7% in 2009–2018, from 0.4% in 1999–2008 to 0.5% in 2009–2018 and from 9.9% in 1999–2008 to 10.7% in 2009–2018 respectively. Poison was observed as the leading means adopted for suicide in India in earlier two decades 1989–1998 and 1999–2008 but suddenly this was replaced by Hanging during the period 2009–2018.

Considering the suicide rate due to different causes viz. family problems, illness, marriage related issues, drug abuse/alcoholic addiction and bankruptcy/indebtedness has increased from 15.5% in 1989–1998 to 23% in 1999–2008, from 18% in 1989–1998 to 22% in 1999–2008, from 2.6% in 1989–1998 to 3.3% in 1999–2008, from 1.2% in 1989–1998 to 1.4% in 1999–2008 and from 1.6% in 1989–1998 to 2.6% in 1999–2008 respectively, while suicides associated with love affairs and ‘Other reasons’ showed a decreasing trend with 4.7% in 1989–1998 to 3.1% in 1999–2008 and from 58.1% in 1989–1998 to 44.3% in 1999–2008 respectively. From the decade 1999–2008 to 2009–2018, all causes show a significant increasing trend of suicide rate (as p<0.0001) except Illness and Other.

Table 2 shows the trends of suicide rates in different States/UTs of India over the last 3 decades. Based on trend patterns of suicide states of India were grouped into four categories viz. the first category with continues rise of suicide rate, the second category with continues decline of suicide rate, a third category whose suicide rate increases first then increases and fourth category whose suicide rate decreases first then increases during past three decades 1989–1998, 1999–2008 and 2009–2018. Hence, we focused on the states belonging to the first and fourth category and those states were Tamil Nadu, Sikkim, Daman & Diu, Madhya Pradesh, Delhi, Gujarat, Haryana, Arunachal Pradesh, Mizoram, Himachal Pradesh, Meghalaya, Punjab, Manipur, Jammu & Kashmir, Lakshadweep, Chhattisgarh, and Jharkhand with an increased decadal growth rate of suicide 16.51%, 64.42%, 8.22%, 22.38%, 25.77%, 18.87%, 18.24%, 24.84%, 64.07%, 35.24%, 145.23%, 35.03%, 8.15%, 45.93%, 146.23%, 22.78%, and 70.18% respectively. So, overall, rates of suicide in small states constituting the states from the North-eastern regions of India and the other small states including 3 UTs show a major trend with an increased rate of suicide.

**Table 2:** Trends of suicide rates in different states/UTs of India over last 3 decades.

States/UTs	Average Decadal Suicidal Rate			Percent change in Average Decadal Suicidal Rate	
	1989–1998	1999–2008	2009–2018	First two Decade	Last two Decade
Puducherry	63.18	52.99	40.07	-16.13	-24.38
Andaman & Nicobar	39.82	34.90	31.61	-12.36	-9.43
Kerala	26.75	28.22	23.73	5.50	-15.91
D&N Haveli	23.58	22.63	19.15	-4.03	-15.38
Goa	19.65	18.86	16.72	-4.02	-11.35
Tripura	19.20	23.46	20.23	22.19	-13.77
Karnataka	19.10	22.32	19.08	16.86	-14.52
West Bengal	17.85	16.98	15.48	-4.87	-8.83
Tamil Nadu	15.52	19.14	22.30	23.32	16.51
Maharashtra	12.34	14.61	14.21	18.40	-2.74
Sikkim	11.83	21.53	35.40	81.99	64.42
Daman &Diu	11.20	10.59	11.46	-5.45	8.22
Assam	10.53	9.97	9.41	-5.32	-5.62
Andhra Pradesh	10.21	15.69	14.39	53.67	-8.29
Madhya Pradesh	9.91	10.77	13.18	8.68	22.38
Odisha	9.35	11.12	10.82	18.93	-2.70
Delhi	8.54	8.11	10.20	-5.04	25.77
Gujarat	8.17	9.59	11.40	17.38	18.87
Chandigarh	7.86	8.44	7.99	7.38	-5.33
Haryana	7.50	10.25	12.12	36.67	18.24
Arunachal Pradesh	6.23	7.81	9.75	25.36	24.84
Rajasthan	5.25	6.53	6.21	24.38	-4.90
Mizoram	4.29	5.40	8.86	25.87	64.07
Himachal Pradesh	3.55	6.13	8.29	72.68	35.24
Meghalaya	3.42	2.83	6.94	-17.25	145.23
Uttar Pradesh	2.82	2.26	2.03	-19.86	-10.18
Punjab	2.60	2.94	3.97	13.08	35.03
Nagaland	2.12	1.43	1.27	-32.55	-11.19
Manipur	1.67	1.35	1.46	-19.16	8.15
Bihar	1.42	0.85	0.74	-40.14	-12.94
Jammu &Kashmir	0.74	1.72	2.51	132.43	45.93
Lakshadweep	0.52	1.06	2.61	103.85	146.23
Uttarakhand	*NA*	3.26	3.20	*NA*	-1.84
Chhattisgarh	*NA*	19.97	24.52	*NA*	22.78
Jharkhand	*NA*	2.18	3.71	*NA*	70.18
Telangana	*NA*	*NA*	24.12	*NA*	*NA*

**Fig 1:**
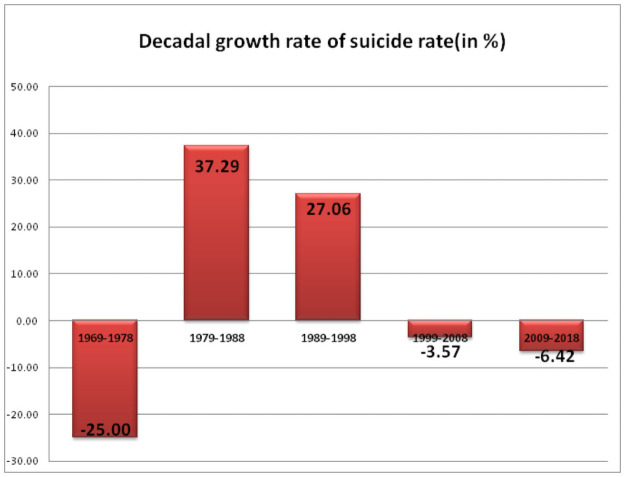
Decadal growth of suicide rate in India (1969–2018).

Fig 1 shows the decadal growth rate of suicide in India; it shows that the suicide rate has decreased by 25% during the early decade of 1969–1978; whereas it increased in the next two consecutive decades 1979–1988 and 1989–1998 with 37.29% and 27.06% respectively. However, it has decreased by 3.57% and 6.42% during the last two decades 1999–2008 and 2009–2018 respectively.

**Fig 2:**
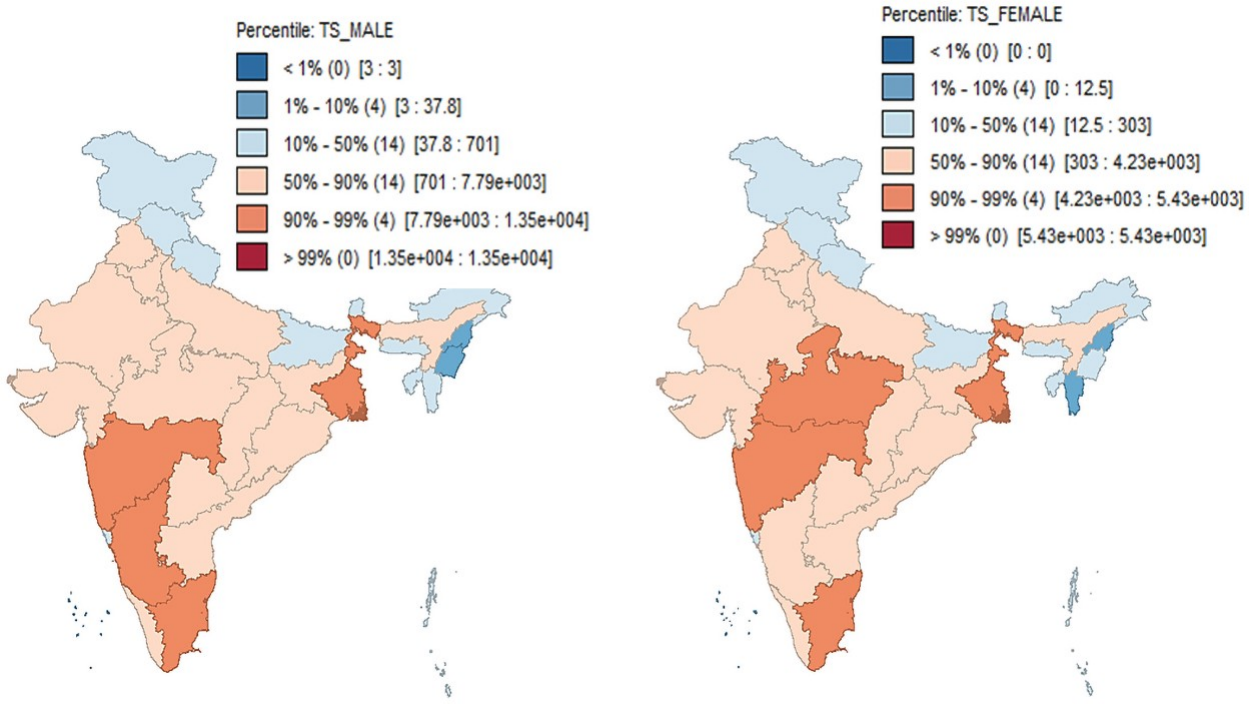
Percentile distribution of total male (left) and female (right) suicide incidences in India for the year 2018.

The Fig 2 shows the percentile distributions of total suicides for both males (left) and females (right) in India for the year 2018. High suicide with more than 90 percentiles can be observed in some states in the eastern, western, and southern part of India for both males and females. The states like Maharashtra, Tamil Nadu, and West Bengal are having consistent high suicidal deaths for both Males and Females, whereas, Karnataka is observed under high suicide for males only; Madhya Pradesh is observed under high suicide for females only. On the other hand, low suicide including less than 10 percentiles can be observed in few states in North-eastern and states in northern parts of India for both males and females. The states viz., Daman & Diu, Lakshadweep, and Nagaland are reporting low suicidal death for both Males and Females. Whereas, Manipur is under low suicide for males only and Mizoram is under low suicide for females only.

**Fig 3:**
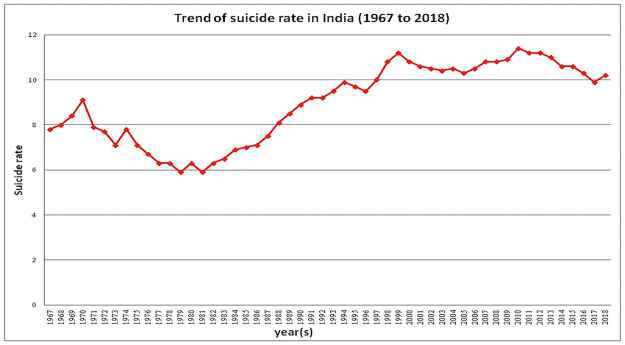
Trend of suicide rate in India (from the year 1969 to year 2018).

**Fig 4:**
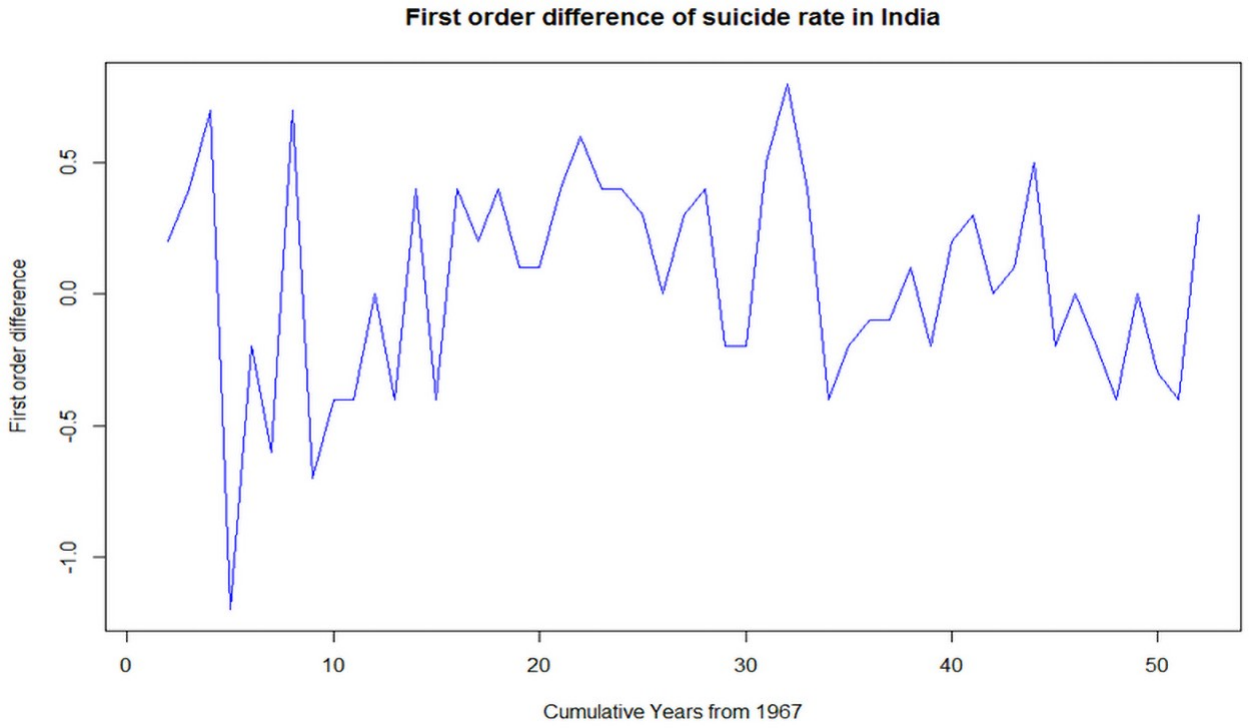
First order difference of suicide rate in India.

Fig 3 highlights the Suicide rate in India with two major picks; a major fall with a decreasing trend up to 1981 followed by a reverse pick with a rising trend up to 1999. For the next couple of decades from 2000 to 2018, the suicide rate has shown a relatively mixed but high incident trend, (during these two decades, the lowest suicide rate was observed in 2017, i.e., 9.9 cases per 100000 population). It can be observed that the trend of suicide rate is non-stationary in nature as its Dickey-Fuller test is not significant with test statistic t = -2.77 and p-value = 0.27. So, we have taken its first differencing as shown in Fig 4, to reduce the trend and stabilize the mean to make the suicide rate stationary. Therefore, we got the first difference is stationary in nature as its Dickey-Fuller test is significant at a 5% level of significance with test statistic t = -3.78 and p-value = 0.03.

**Fig 5:**
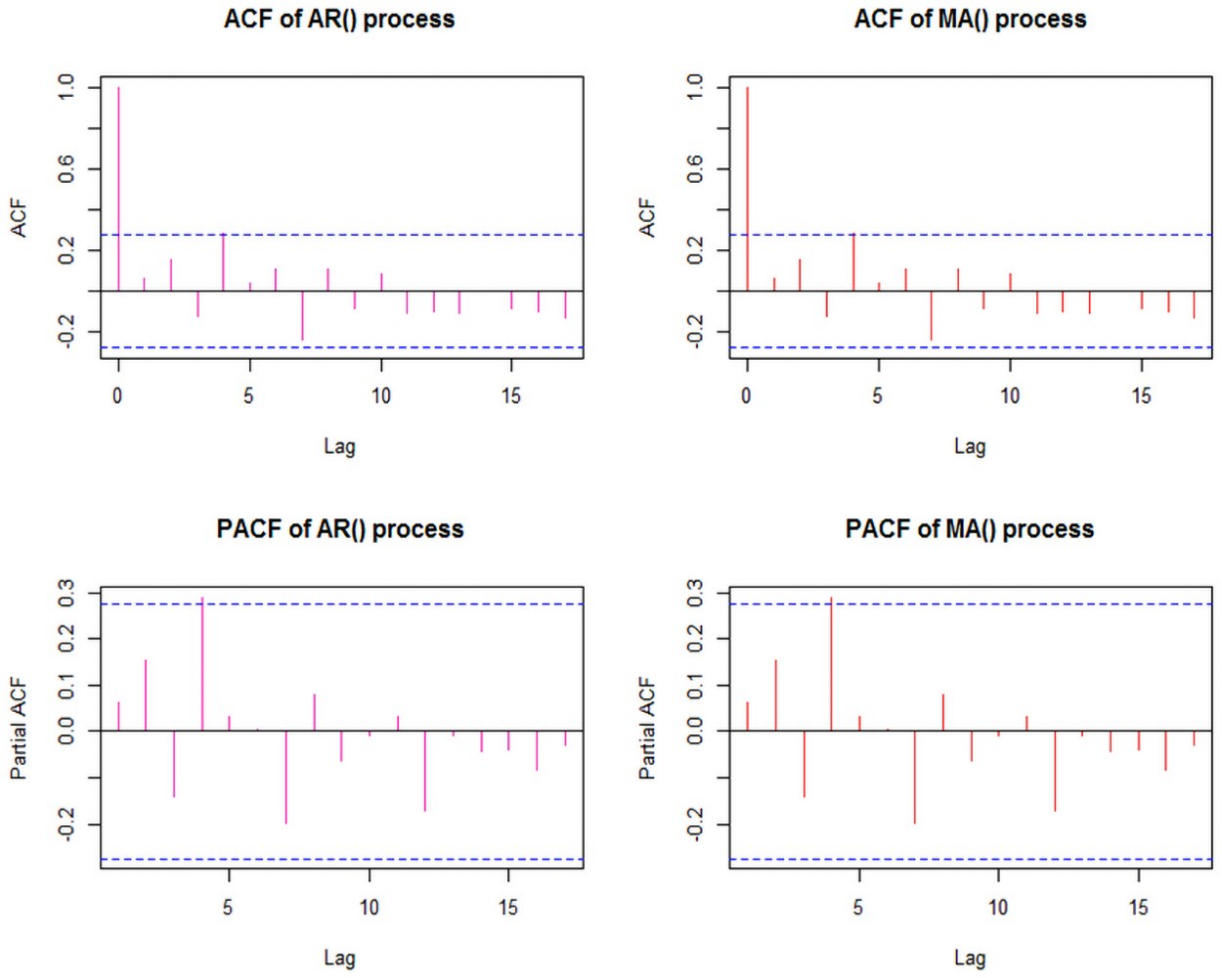
ACF and PACF plot of suicide rate in India.

**Fig 6:**
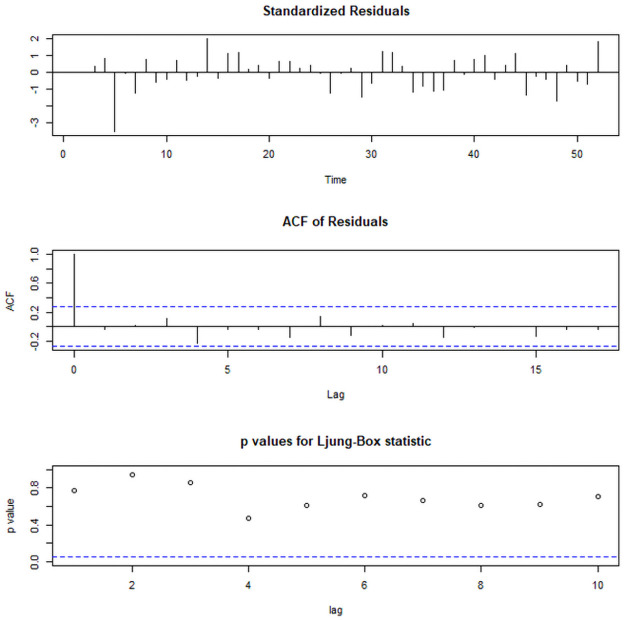
Plots for residual and p-values of Ljung-Box statistic.

**Fig 7:**
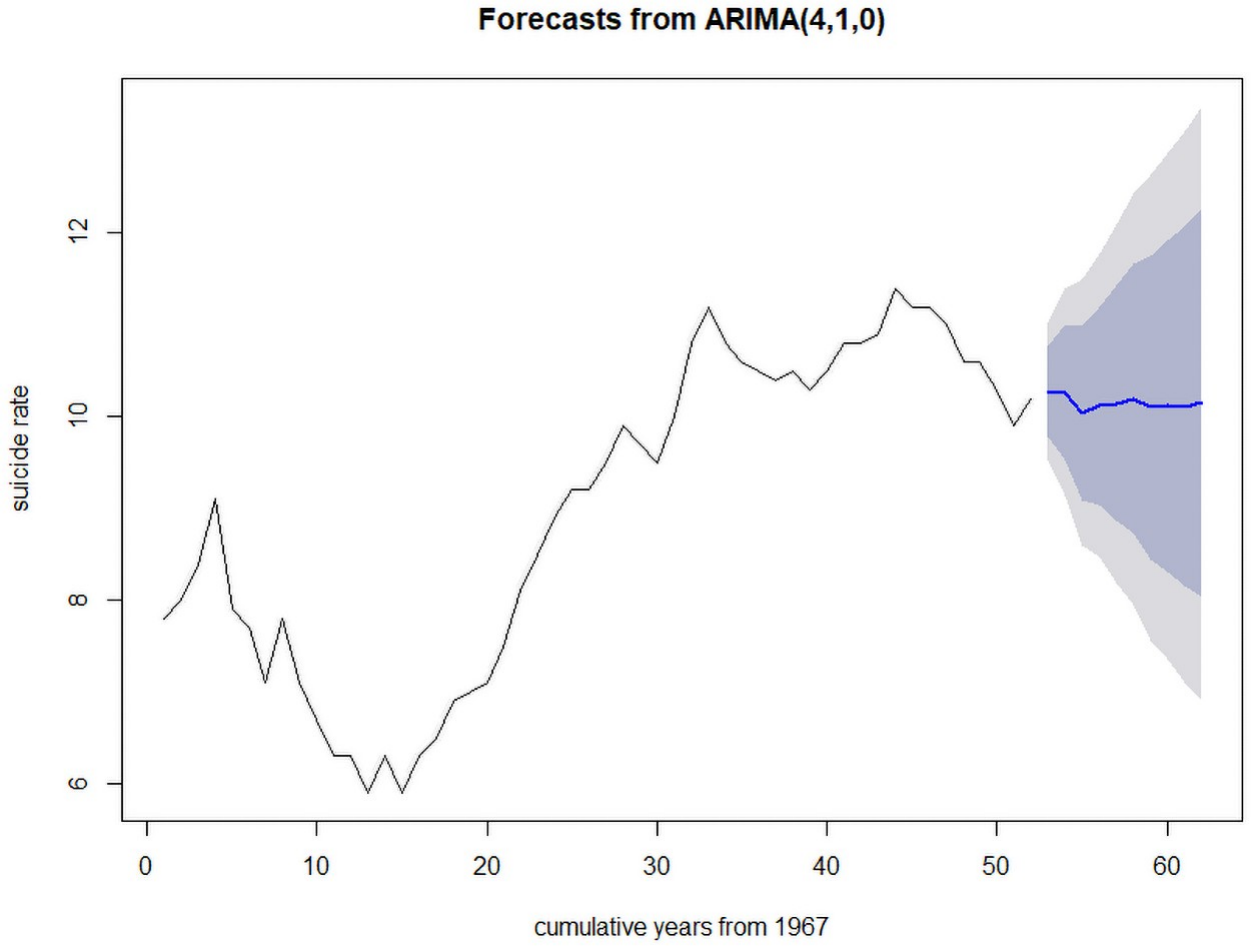
Forecasted plot of the suicide rate for India with 95% CI.

As we have identified the degree of ARIMA model (i.e., d = 1), from ACF and PACF plot (Fig 5) the following ARIMA models have been suggested to estimate other parameters of the model: ARIMA(4,1,4), ARIMA(2,1,4), ARIMA(4,1,2), ARIMA(2,1,2), ARIMA(4,1,0), ARIMA(0,1,4). The ARIMA(4,1,0) model has been identified as the best fit ARIMA model for suicide rate data of India as its AIC, BIC, and AICc values are lowest among all other suggested models (Table 3). The Box-pierate test (χ^2^ = 0.02, p-vaue = 0.89) states that the best fit model doesn’t show a lack of fit i.e., the ARIMA(4,1,0) model is fine to use. Fig 6 shows the plot for Standardized residuals, ACF of residuals, and p-value of Ljung-Box statistic [26]. The values of estimated parameters with their residuals and significance of the best fit ARIMA(4,1,0) model shown in Table 4. Table 5 shows the forecasted values of suicide rates with its 95% CI for the next 10 years (i.e., 2019–2028). As per our best fit ARIMA(4,1,0) model, the suicide rate in India is expected to be 10.15, more particularly to lie between 6.92 and 13.38 in the next decade. The forecasted trend with its 95% forecasted Interval of the suicide rate for India is shown in Fig 7.

**Table 3:** AIC, BIC and AICc values for suggested ARIMA models for sucide rate in India.

Model	Loglikelihood	AIC	AICc	BIC	ME	RMSE	MAE	MPE	MAPE	MASE	R^2^
ARIMA(4,1,4)	-18.96	55.93	60.32	73.31	0.032	0.330	0.276	0.320	3.208	0.838	0.965
ARIMA(2,1,4)	-21.70	57.40	60.00	70.92	0.035	0.363	0.297	0.333	3.480	0.901	0.958
ARIMA(4,1,2)	-21.24	56.49	59.09	70.01	0.026	0.360	0.307	0.280	3.571	0.933	0.958
ARIMA(2,1,2)	-24.40	58.79	60.13	68.45	0.047	0.376	0.306	0.415	3.664	0.929	0.954
**ARIMA(4,1,0)**	**-21.27**	**52.54**	**53.87**	**62.20**	**0.035**	**0.377**	**0.317**	**0.327**	**3.702**	**0.961**	**0.954**
ARIMA(0,1,4)	-23.28	56.55	57.89	66.21	0.027	0.361	0.308	0.287	3.575	0.934	0.958

**Table 4:** Parameters of best fit ARIMA (4,1,0) model.

Parameters	Coefficients	Std. error	t-statistic	P-value
AR1	0.1394	0.1296	1.08	0.001
AR2	0.0694	0.1291	0.54	0.003
AR3	-0.2028	0.1341	-1.51	0.012
AR4	0.4079	0.1489	2.74	0.023

**Table 5:** Forecasted value of suicide rate for next 10 years based on ARIMA(4,1,0) model with 95% confidence interval.

*Year*	*Forecasted value*	*Lower 95% CI*	*Upper 95% CI*
2019	10.27487	9.531394	11.01834
2020	10.26487	9.137789	11.39195
2021	10.04468	8.594108	11.49526
2022	10.12049	8.474072	11.76691
2023	10.14833	8.197248	12.09942
2024	10.19805	7.956360	12.43974
2025	10.10172	7.566300	12.63714
2026	10.11703	7.356995	12.87706
2027	10.11375	7.111683	13.11581
2028	10.15416	6.923429	13.38490

## Discussion

In this paper, we have tried to study the trends of suicide in India by analyzing past decades data and forecasting for the next decade; along with we have examined the patterns of distribution of different important attributes of suicide viz. gender, age group, education, profession, social status, means adopted and causes of suicide over last three decades. Finally, we have discussed selected broader perspectives based on various previous observations and evidence to highlight the socio-cultural implication and policy perspectives in the context of rising incidences of suicide in India. We have done this analysis with great care; in our analysis, we found the trend where the suicide rate in India was highly fluctuating. It can be observed (Fig 3) that trend of suicide in India experienced a decline from the year 1970 to 1980 with a slight rise in the year 1974; however, the rate of suicide continued to rise from 1981 through 1990 and reported the highest number of suicide in the year 2000- the maximum in number till date; from the year 2000 onwards the rate of suicide marginally declined, with a further rise from 2006 onwards with the pick in the number of cases of suicide during the years 2010. The trends of suicide post-2011 though showed a pattern indicating a fall, it again took a rise towards the end of the decade i.e the year 2018. It was observed of increasing from 9.9 per 100,000 in 2017 to 10.2 per 100,000 in 2018. Thus we can expect an increasing trend for the future, which is found in ARIMA (4,1,0) model in our analysis and we have found the same pattern in a recently published suicide report by NCRB where it is increased to 10.4 per 100,000 in 2019. Our findings indicate the trends of suicide among males and females co-varies over years (Fig 2) unlike industrialized nations; similar observations were also reported previously [27]. Several important trends were observed in the last three decades’ data. States like Maharashtra, Tamil Nadu, Karnataka, and West Bengal are the states with major numbers of incidences of suicides along with showing a trend of consistently high numbers of suicidal deaths for both Males and Females. While N-E states and several other small states including the UTs have shown a trend of lesser suicide in terms of percentile mainly due to their comparatively small size, these states have shown a rising trend of suicide during the period of study; states like Sikkim, Mizoram, Meghalaya, Arunachal Pradesh, Manipur, other small states like Punjab, Himachal Pradesh, Haryana and UTs like Delhi, Lakshadweep, and Jammu & Kashmir are showing a rise in the suicide rate during the recent last decade than the previous. There are four UTs particularly belonging from the southern side of India among the top 5 suicide reporting states/UTs. Simultaneously, other southern states (Kerala, Karnataka, Tamil Nadu) were also observed with high incidences. On the rising side, states like Sikkim (64.07%), Meghalaya (145.23%), Mizoram, Lakshadweep (146.23%), Jammu and Kashmir (45.93%), and Jharkhand (70.18%) have reported a significantly high percentage rise in suicide during last two decades which is a real concern (Table 2). A high rise in suicide was also observed in certain other states like Madhya Pradesh, Gujarat, Jharkhand, and Chhattisgarh. Such rising trends of suicide need further detail and longitudinal studies around the causes and associated factors for such a rising trend of suicide to take necessary control measures.

**Fig 8:**
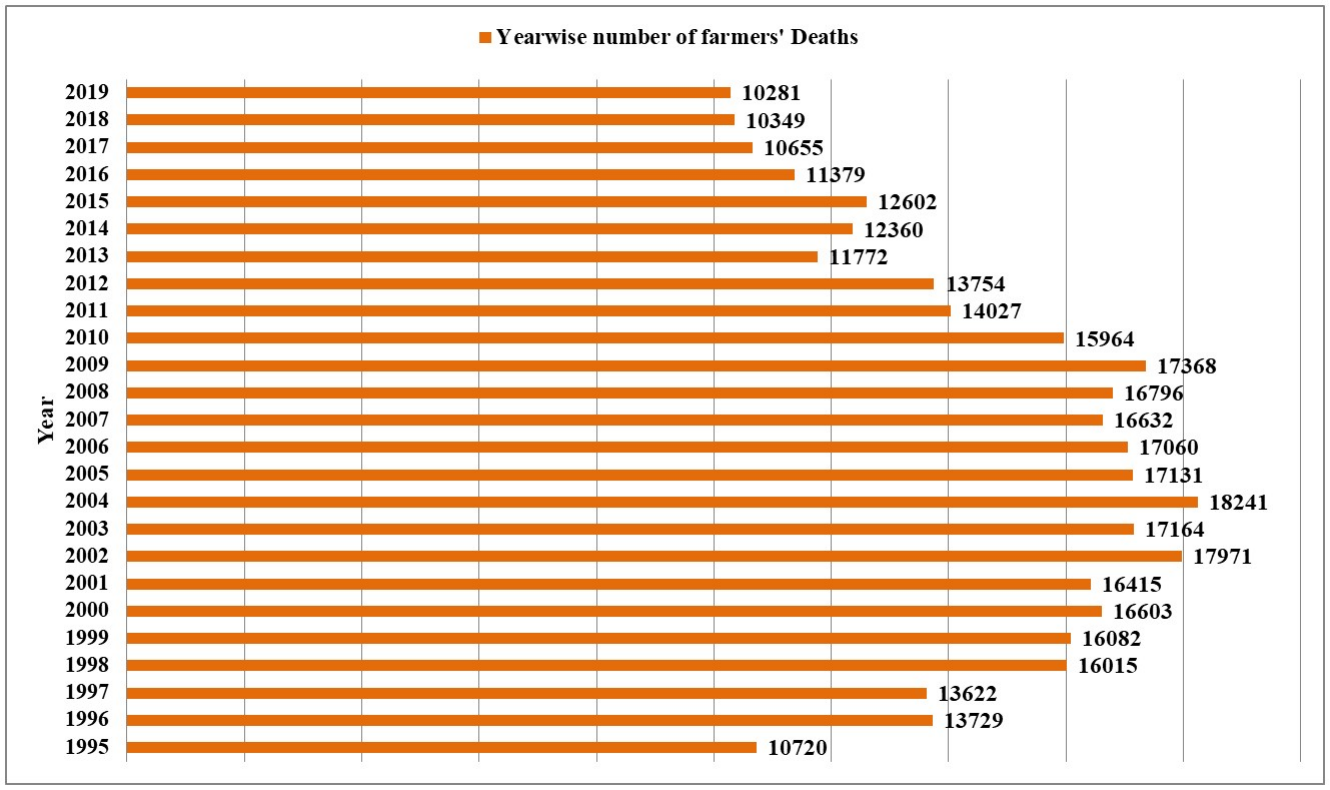
Year-wise numbers of farmers’ suicidal deaths in India during 1995–2019.

Considering major attributes of suicide, it may be mentioned here that most of the studies in India have examined the socio-demographic and psychological aspects of suicide quantitatively. Similarly, studies examining other aspects like genetic/epigenetic, physiological, or biological basis of suicide risks in India are too scanty. So, in the present context, the limited studies suggest early age group involving adolescents and youths [2,4], gender [3,4], easy availability of lethal insecticides [5], unemployment [6–8], adverse marital status/outcomes for males and females [9], illnesses, psychiatric and personality disorders [10–13] as the major risk factors of suicide. An important trend from the annual data is visible from Fig 3 that post-1990s there is a consistent surge in suicide rate (Fig 8); such rise in suicide incidences during 1990s onwards has mainly been attributed to the liberalization policy in the Indian economy adopted by the government during that time as well as the successive governments causing serious agrarian crisis [28–30]. The issues like the rising cost of agricultural inputs, rising unpaid farm loans burden, lack of facility to sell the agricultural products, failure to get Minimum Selling Price (MSP), lack of compensation to crop loss due to recurring natural calamities like heavy rain, flood, drought, etc. have been reported as driving factors for farmers to take the extreme step to commit suicide. Studies have reported a significant relationship between the percentage of marginal farmers, cash crop production, indebtedness, and suicide rates [31] However, explanations exploring the socio-cultural or psychological pathways of attempt or committing suicide are limited. So, it is imperative at this point to promote research studies to examine the pathways which will immensely help in prevention. At this point, it may be highlighted that socio-psychological reasons like stress and social isolation triggered anxieties have been observed as major causes of suicide in India than clinically psychiatric issues that trigger suicide [32]. So, understanding such pathways will provide useful dimensions and scopes for intervention.

So, in the above context, few facts, based on the present analysis, need to be highlighted here. It was observed, in the last decade of 2009–2018, that the percentage of male suicide victims was 66.2% nearly double as compared to female suicide victims i.e., 33.8% in India. Patel et al. 2012 also found that age-standardized suicide rates per 100000 people aged 15 years and older are 26.3 for men and 17.5 for women [33]. The trends of male-female suicide incidences show that the share of male suicide has significantly increased (from 58.6% to 66.2%) over the periods of last three decades while the incidences for females have decreased substantially from 41.2% to 33.8% during that time. Balint et al.(2020), in a recent analysis on fall in suicide rate in Hungary, found that an increase in educational attainment in the availability of institutional social support has a significant effect on reduction in suicide incidents among both males and females as improvement in education level helps in taking preventive measures against suicide at the population level [17,34,35]. Female education in India during the last three census decades has got 25 points improvement (39.29%,1991 census;53.67%,2001; 64.63%, 2011) [36]; particularly, the interdecadal change is substantial with a jump of about 14% and 12% respectively. On the other hand, male education level had already reached a level (64.13% during 1991 to 80.88% during 2011; [36]) where the expectation of conversation of education to results (employment, income, etc) are high priorities; in this context, it may be mentioned that Indian rural society which lags in terms of access to education and income [36] report double the number of cases of suicide than the urban society which have better access to education and employment [33]. Furthermore, while Indian society is increasingly experiencing steep social inequality and disparity in terms of economic growth, with rising concerns among the citizens for carrier management, income generation, getting a job, and family responsibility like aspects, such factors are becoming major affective issues for men in particular as they are the breadwinners in a majority of the families. India experiences patrilineal family patterns dominating all over, Failure to attain those expectations is possibly a major factor in the rise in suicide among males. Similar findings are also evident from a study in South Korea by Kim et al 2017 which shows the male suicide rate as three times higher than that of females [1]. Here, it is at the same time happening that the modernization influenced changing marital practices, marital status and post-marriage life are counterbalancing the preliminary expectations [37,38]; education is also playing an important role [37]. In the context of the high patriarchy, this counterbalance is primarily breaking the established male supremacy by affecting males of India and possibly leading to a rise in the suicide rate among them in the post-marital period; similar observations were also made by Steen and Meyer. (2004) [37].

On the front of means of suicide, the suicide rate increased in the last two decades only for the three categories like Hanging, Self-inflicting injury, and Other means. Poisoning was the leading means adopted for suicide in India in the two decades 1989–1998 and 1999–2008 but suddenly this was replaced by Hanging from 2009–2018 onwards. It is reasoned that while agricultural pesticides previously being used as the primary and major source of poison for committing suicide, with increasing awareness around this aspect and changing patterns of pesticide use, there has been a visible fall in such hazardous practice.

Furthermore, beyond specific aspects, we have tried to explain the possible risks of an increase in the overall suicide rate based on various pieces of evidence and hypotheses.

### India’s state of human development and trends in suicide

A consistent association between the Human Development Index (HDI) and a rise in rates of suicide has been observed [24,39,40] in recent times. HDI takes the aspects of health, education, income, life expectancy, adult literacy, years of schooling, equitable distribution of income, GDP for purchase power parity, and gender parity into consideration. In the last decade, India has experienced a dismaying scenario regarding HDI along with further falls within that, which might have a significant role in rising incidences of suicide cases. Furthermore, the gender parity index of India is significantly low and it is lower than the world average during this period which further explains the possible comparatively high suicidal rate among women in India than other parts of the world. All these parameters individually and collectively may cause distress at least among a significant section of the population to take an ultimate decision to give up in life. However, such generalized indexes need to be taken with caution as they alone cannot explain the reasons for suicide. As, in a paradoxical finding, some other Indian studies have reported people experiencing higher GDP, industrial growth, and well-off in their lives are also at increased risk of suicide [41,42]. Furthermore, an increased rate of suicide among adolescents and youths is also another concern. As no such age and sex, detailed data is available in this regard, it needs thorough exploration to understand suicide in the context of India which is highly diverse in its social, economic, and cultural spaces.

### High incidences of illness associated suicide

In the present analysis around suicide in India, we would highlight the high incidences of suicides around illness over the whole span of the period of analysis. As it can be observed from Table 1, the percentage of suicide contributed to illness during the periods 1989–1998, 1999–2008 and 2009–2018 are 18.0%, 22.0%, and 18.5% respectively. Here, it can be mentioned that such suicides contribute the second-highest number of cases in the last three decades among all known causes of suicide after ‘Family problems’. In recent times, several diseases like cancer, diabetes, cardiovascular diseases, pollution associated health problems like issues that are requiring expensive and long term medical care have been observed with a significant rise in their occurrence; poor having no or little affordability are expected to be the major sufferers of such health hazards; on the other hand, infectious diseases like tuberculosis, leprosy, etc with the serious social stigma attached and mostly affecting economically underprivileged are increasing or remerging. At an overall level, the burden of illness while becoming overwhelming over time, the public healthcare has evolved unequally to such burden to accommodate the increasing healthcare needs particularly of the underprivileged section that constitute a major part of the Indian population and primarily dependent on such public healthcare facilities. Positioning it in the context of recent findings, a study on the suicide rate in European Union has reported that the proportion of expenditure on health of total government expenditure was one of the strongest predictors for the rise in suicide in the population around health issues [43]. In similar another observation, lack of access to healthcare- inclusive of mental health care has also been observed as a contributing factor to rising risks of suicide in the USA [44]. In developing countries like India, mental health services are mostly neglected or unavailable [45] on one end while on the other side, are becoming highly expensive, mainly at the private facility level; notwithstanding the availability of facilities, seeking such services is highly stigmatized in Indian society. So, while India is aspiring for economic growth and developmental expansions, high social inequality and economic disparities are also well evident; policymakers and health care administrators must consider the allocation of resources to address rising public health care needs, health of the poor, and most importantly, the associated mental health care needs [44].

### Emile Durkheim’s organic theory of suicide [38]

We would further juxtapose some theoretical perspectives to the present context from an Indian societal functional point of view. India’s more than 60% population lives in villages with mostly traditional social patterns. So, various cultural, moral, and social- institutional aspects have important bearings on such individuals subscribing to those traditional views. Contextualizing India’s increasing rate of suicide in the theoretical framework of the Social theory of suicide [38] it can be observed that suicide is more organic which, as per Durkheim, can occur to anyone in any society. In his seminal work ‘La Suicide; Etude de sociologies (1897)’ (Suicide: A study of Sociology), Emily Durkheim provided a social and cultural explanation to suicide with empiricism where he explained the idea of suicide from the perspectives of the division of labor, social constraints and collective conscience [46]. While suicides are regular in their occurrence in any society, any increase in its rate is an explanatory index for increasing disintegrating forces at work in the social structure with ramifying functions in that concerned society. Such negative conditions cause three major acts: Inducement, Perpetuation, and Aggravation of suicidal potential. Durkheim in this context put suicide in four different contexts: A) Egoistic suicide where the individual lacks altruistic feelings and feels socially isolated and realizes that he has no place in the society and therefore destroys self; this is a most common reason that contributes to the highest cases of suicide in India. Here it may be mentioned that suicide due to illness, diseases, discrimination around caste, identity, etc, and the largest portion of suicidal cases in India fall within this type of suicide; B) Altruistic suicide where excessive binding on the individual with the moral order of the society leading to suicide; this is a common phenomenon among adolescent suicide in India. Similar observations are also there around suicide for failure in love affairs, loss of honor leading to suicide at the mass family level, happening at a concerning rate in the India scenario. Another such pattern i.e. self-immolation constitutes more than 10% of the methods of suicide in India; C) Anomic (normlessness) suicide where normative frameworks or regulative power in a society are disrupted at the collective level, positively or negatively causing, for example, poverty or crises of prosperity. It is highly observed in Indian scenario where suicide happens as a result of poverty, non-affordability, workplace harassments, failure to achieve rightful due as well as among the celebrities and well-up families; D) Fatalistic suicide where high or extreme of the regulation continuum happens which Durkheim further explains as “persons with futures “pitilessly blocked and passions violently choked by oppressive discipline”. Examples of such suicide in the Indian context are observed in incidences of increasing suicide by young husbands and wives, children of excessively conservative parents, etc.

In this context, the ‘social integration’ [38] at various levels like community/society/family has an important role in addressing suicide incidences; an increase in disruption leads to depression and loneliness among the members which over time brings a rise in suicide in the population [46]. So, efforts towards an inclusive society, facilitating social care, and encouraging engaging communities and social cohesion will have lasting effects on the population towards suicide prevention. Along with, promotion and encouragement for various support groups at social, cultural, and institutional levels, community empowerment with mental health support, increasing community-based support facilities with trained counselors will have visible results on control of suicide incidents. Most importantly, focused measures around the de-stigmatization of mental health issues are highly important. Addressing achievable goals in minimizing social disparities in various social, economic, and demographic indexes like gender, education, access to health care will have positive bearings on suicide control.

### Understanding the bottlenecks

Suicide remained a criminal act and a non-cognizable offense under Indian Penal code 309 up to the year 2018 until it was decriminalized by a bill passed in both lower and upper houses of India [47]. Similarly, there are strongly attached socio-cultural stigma and negative attitudes towards seeking help and institutional support for mental health issues including depression, which are major challenges in suicide prevention in India. Such a scenario further makes it nonconductive to take necessary steps and measures to minimize the incidences of suicide [48]., Additionally, there is no such regulation except a guideline (provided in the year 2019) on sensitive and proper reporting on suicide in public places, electronic or print media as such mediums of reporting suicide to have significant influences in increasing suicide [49]. Researches around suicide in India, its risk factors, social, cultural, and psychological as well as biological association and pathways have been limited in holistically exploring the problem while they have been pursued more in epidemiological and cross-sectional mode. So, evidence is less or limited regarding ‘how and what to address for controlling suicide’. Considering the high diversity in socio-cultural and cognitive perspectives in various societies in India which is further stratified by multiple layers with distinct features like ethnicity, locality, economic status, and social strata, it needs to develop and implement suicide control practices complementary to the particular society under consideration.

Furthermore, the long-standing issue of the suicide rate among various vulnerable and indigenous communities, tribal populations have less been pursued along with limited reports. Considering the national trend of the annual suicide rate at 0.9% in comparison to 1.2% at the world level, it is necessary to map such a status among the tribes. Elwin, in the year 1950 describing 250 tribal suicide cases highlighted the alarming state of high suicide tendency among tribes of Bastar in Chhattisgarh and Madhya Pradesh [50]. Similarly, Nane (2013) observing the history and present state of mental health among Idu Mismi tribes in Arunanchal Pradesh reported the high vulnerability of the community towards mental health problems with the alarming rate of the suicidal tendency among the tribe [51]. Similar findings have been reported among other tribes [52]. It is important to note that there is no such systematic reporting of mental health problems and the prevalence of suicide from tribal communities. For example, Nane (2013) in his findings on suicide among Idu Mismi observed only 2 cases in a government report from among more than 250 cases that he recorded during his research [51]. So, in the rapidly changing tribal socio-cultural scenario when these indigenous groups are readily embracing modernity, such traditional wisdom that is limited to the idea of the absence of mental health issues among them, needs rethinking.

So suicide preventive measures in India have huge scope to further improve. Considering the aspect of increased suicides as a result of illness as reported in NCRB reports from time to time as a 2^nd^ major reason for suicide, it is highly important to formulate appropriate and inclusive health plans and policies to help citizens to address their illnesses successfully; government support in healthcare by making it affordable and accessible can play a pivotal role in this regard. The limited scope for addressing the mental health of children and adolescents under various national health programs like Rashtriya Bal Swastya Karyakram, Ayushman Bharat as well as under various statewide health programs need to be more inclusive regarding mental health issues like depression, anxieties among children, adolescents and youths. Elderly mental health needs focus in general healthcare programs as elder people in Indian society are highly neglected due to their limited economic contribution to family, several traditional wisdom like mental health is an old age issue and need not worry, etc. Individualized therapy, group/community therapy, and family-based psychological education like facilities along with institutional medication and clinical care will be a step forward for a holistic approach for improving mental health [53]. Timely review, evaluation, and appropriate modification in existing policies and by bringing in new policies in different sectors like farming and agriculture will also add to address public agony and concerns that further manifests and becomes acute at the individual level to take the extreme step of taking life. Addressing socio-cultural stigma and negative attitudes towards mental health issues and associated health-seeking behavior need long-term, consistent and appropriate measures.

It is apt to mention here that we cannot prevent all the suicide deaths [38] but we can devise some strategies that will reduce suicidal risks. A multi-sectoral prevention approach may help in the direction. Thus, this research will be helpful for the policymakers, program implementers, social advocacy groups to put their efforts to reduce the suicide rate among the population at risk.

### Limitations of the study

It is worth here mentioning the limitations of our study. We have used secondary data i.e., NCRB record for this analysis it has its limitations like under-reporting. In India suicide is considered a crime hence it may affect the veracity of reporting. Thus, these results should be interpreted with caution.

## Supporting information

S1 Table (XLSX)

S1 File (PDF)
