# Supplementary material for: Forecasting suicide rates in India: An empirical exposition
Source: PLoS One. 2021 Jul 29;16(7):e0255342. doi: 10.1371/journal.pone.0255342 (PMC8321128; doi:10.1371/journal.pone.0255342)
Supplement: S1 File — (PDF) [file pone.0255342.s002.pdf]

CHAPTER – 2

SUICIDES IN INDIA

Each suicide is a personal tragedy that prematurely takes the life of an individual and has a continuing ripple effect, dramatically affecting the lives of families, friends and communities. Every year, more than 1,00,000 people commit suicide in our country. There are various causes of suicides like professional/career problems, sense of isolation, abuse, violence, family problems, mental disorders, addiction to alcohol, financial loss, chronic pain etc. NCRB collects data on suicides from police recorded suicide cases.

Rate of suicides has been calculated using projected population for the non-census years whereas for the census year 2011, the population of the Population Census 2011 was used.

A total of 1,34,516 suicides were reported in the country during 2018 showing an increase of 3.6% in comparison to 2017 and the rate of suicides has increased by 0.3 during 2018 over 2017. [**Table–2 (A)**] The incidence and rate of suicides during 2008-2018 is presented in **Table-2.1**.

Table – 2 (A)

Number of Suicides, Growth of Population and Rate of Suicides during 2014 - 2018

Sl. No.	Year	Total Number of Suicides	Mid-Year Projected Population* (in Lakh)+	Rate of Suicides*** (Col.3/Col.4)
(1)	(2)	(3)	(4)	(5)
1	2014	1,31,666	12,440.4	10.6
2	2015	1,33,623	12,591.1	10.6
3	2016	1,31,008	12,739.9	10.3
4	2017	1,29,887	13091.6#	9.9
5	2018	1,34,516	13233.8#	10.2

* Source: The Registrar General of India.
\# Source: Report of the Technical Group on Population Projections (November, 2019), National Commission on Population, Ministry of Health & Family Welfare.
\+ One Lakh = 0.1 Million
*** Rate of Suicides = Incidence of suicides per one lakh (1,00,000) of population.
- As per data provided by States/UTs.

Number and Percentage Share of Suicides in States/UTs

The State/UT and City wise information on the incidents of suicides, its percentage share in total suicides and rate of suicides during the year are presented in **Table–2.2**.

Majority of suicides were reported in Maharashtra (17,972) followed by 13,896 suicides in Tamil Nadu, 13,255 suicides in West Bengal, 11,775 suicides in Madhya Pradesh and 11,561 suicides in Karnataka accounting for 13.4%, 10.3%, 9.9%, 8.8% and 8.6% of total suicides respectively. These 5 States together accounted for 50.9% of the total suicides reported in the country. The remaining 49.1% suicides were reported in the remaining 24 States and 7 UTs. Uttar Pradesh, the most populous State (16.9% share of country population) has reported comparatively lower percentage share of suicidal deaths, accounting for only 3.6% of the total suicides reported in the country.

The States which have witnessed significantly higher number of suicidal deaths during the year 2016 to 2018 are presented in the **Table–2(B).** These States have continuously accounted for about 7.0% or more of the total suicides reported in the country from 2016 to 2018.

Figure – 2.1

State/UT wise Major Percentage Share of Suicides in States during 2018

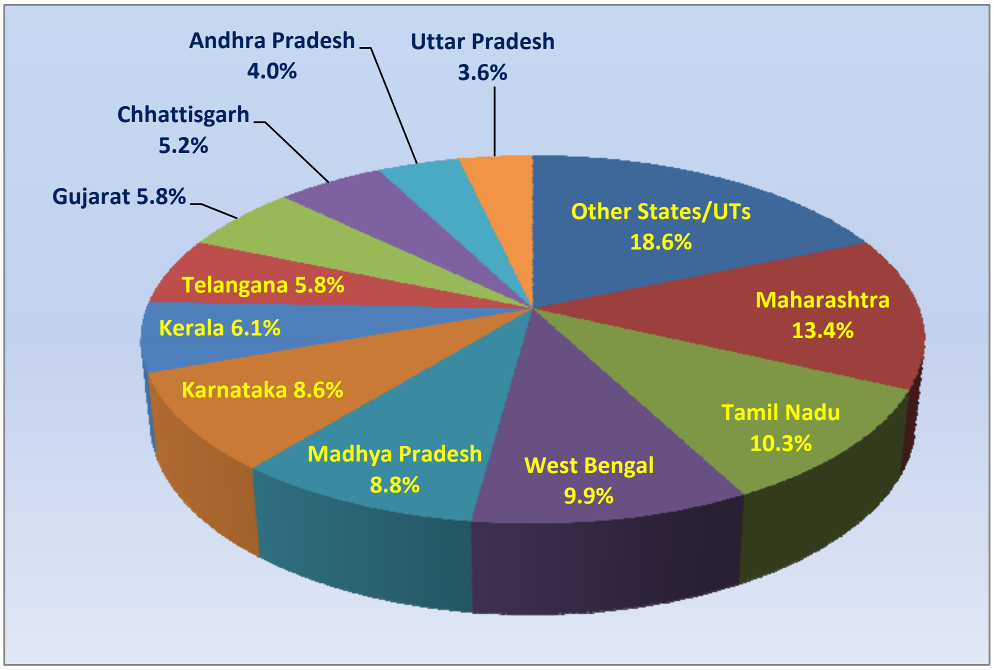

- As per data provided by States/UTs.

Note: OTHER STATES/UTs include Arunachal Pradesh, Assam, Bihar, Goa, Haryana, Himachal Pradesh, Jammu & Kashmir, Jharkhand, Manipur, Mizoram, Nagaland, Meghalaya, Punjab, Rajasthan, Sikkim, Tripura, Odisha, Uttarakhand, A & N Island, Chandigarh, D & N Haveli, Daman & Diu, Delhi, Lakshadweep and Puducherry

STATE/UT – WISE TOTAL NUMBER OF SUICIDES DURING 2018

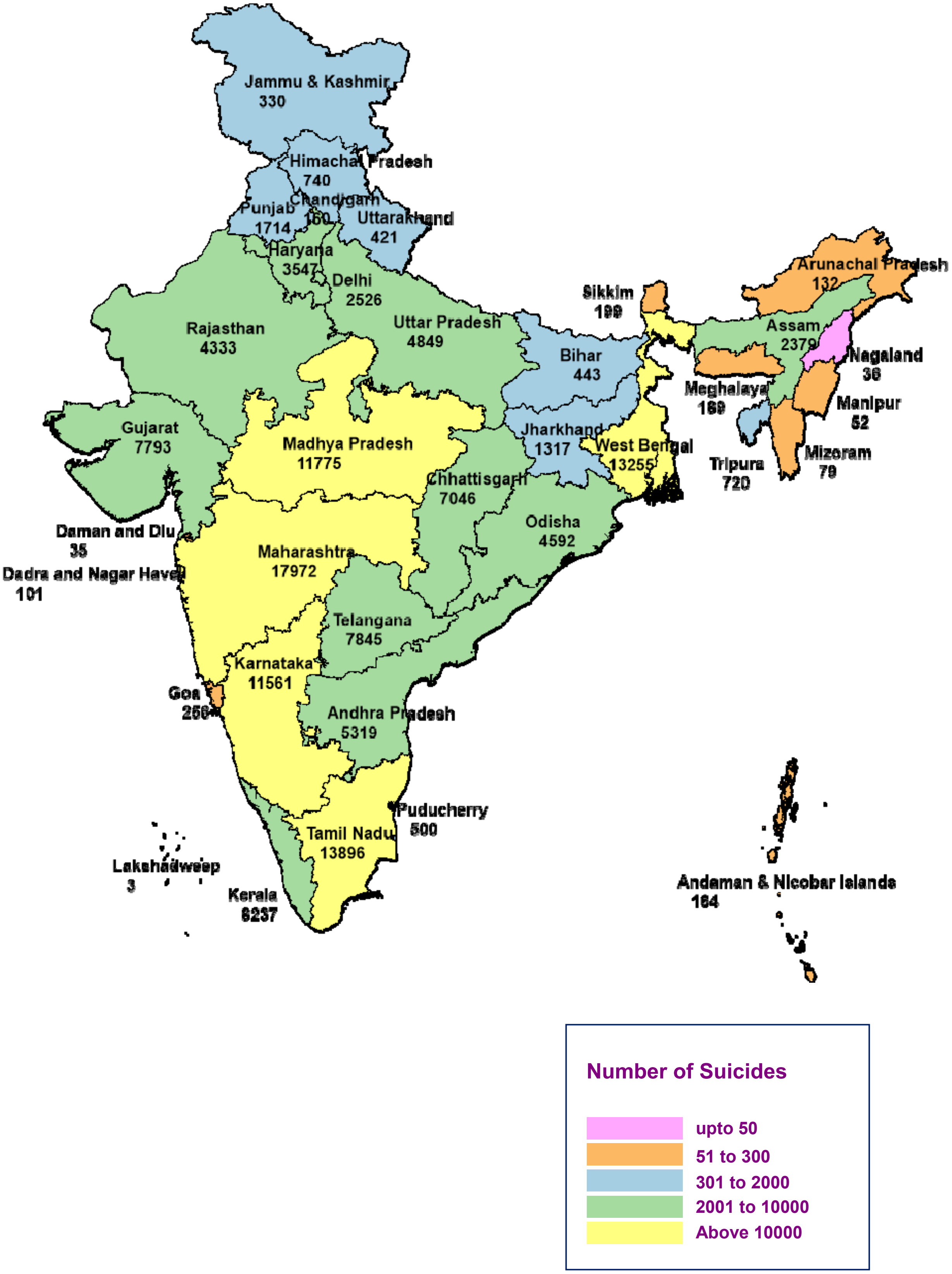

Map Powered by DevInfo, UNICEF

- As per data provided by States/UTs.

Table – 2 (B)
States with Higher Percentage Share of Suicides during 2016 to 2018

Sl. No.	Year					
	2016		2017		2018	
1	Maharashtra	(13.1%)	Maharashtra	(13.6%)	Maharashtra	(13.4%)
2	Tamil Nadu	(11.6%)	Tamil Nadu	(11.1%)	Tamil Nadu	(10.3%)
3	West Bengal	(10.3%)	West Bengal	(9.2%)	West Bengal	(9.9%)
4	Karnataka	(8.2%)	Madhya Pradesh	(9.1%)	Madhya Pradesh	(8.8%)
5	Madhya Pradesh	(8.0%)	Karnataka	(9.0%)	Karnataka	(8.6%)

- As per data provided by States/UTs.

Delhi, which is the most-populous UT, has reported the highest number of suicides (2,526) among UTs, followed by Puducherry (500). Seven UTs together accounted for 2.6% of total suicides in the country. A total of 21,408 suicides were reported in the 53 mega cities of the country during the 2018.

The States and UTs which have reported significant percentage increase in suicides in 2018 over 2017 were Arunachal Pradesh (48.3%), Chandigarh (41.6%), D & N Haveli (34.7%), Himachal Pradesh (28.5%), Uttarakhand (27.2%) and Manipur (20.9%) while highest percentage decrease was reported in Daman & Diu (14.6%), Puducherry (13.9%), Mizoram (13.2%), Sikkim (7.0%) and Goa (6.2%) **[Table–2.3]**.

Rate of Suicides — Trend in States/UTs

Rate of suicides i.e. the number of suicides per one lakh population, has been widely accepted as a standard yardstick for comparison. All India rate of suicides was 10.2 during the year 2018. A & N Islands reported the highest rate of suicide (41.0) followed by Puducherry (33.8), Sikkim (30.2), Chhattisgarh (24.7) and Kerala (23.5). The details of States/UTs which have recorded higher rate of suicides during 2016 to 2018 are given in **Table–2(C)**.

Figure – 2.2

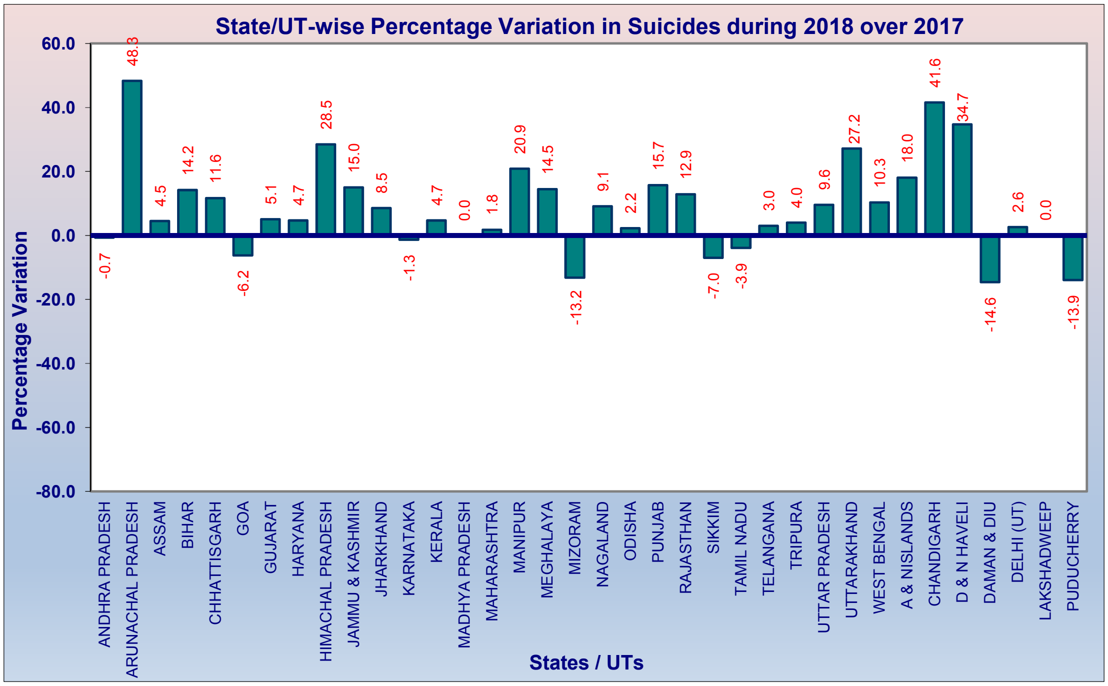

- As per data provided by States/UTs.

Figure – 2.3

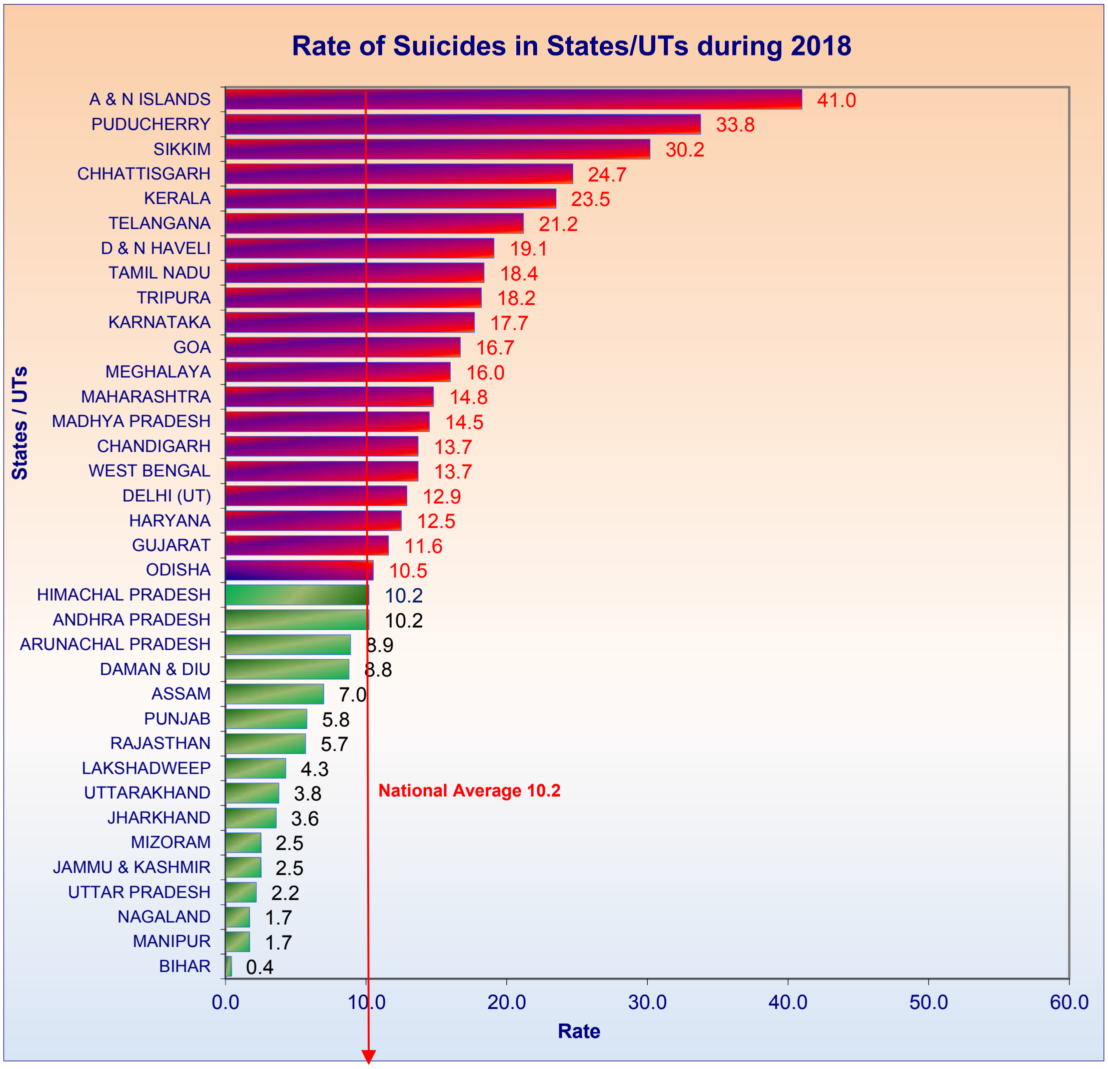

- As per data provided by States/UTs.

STATE/UT - WISE SUICIDE RATE DURING 2018

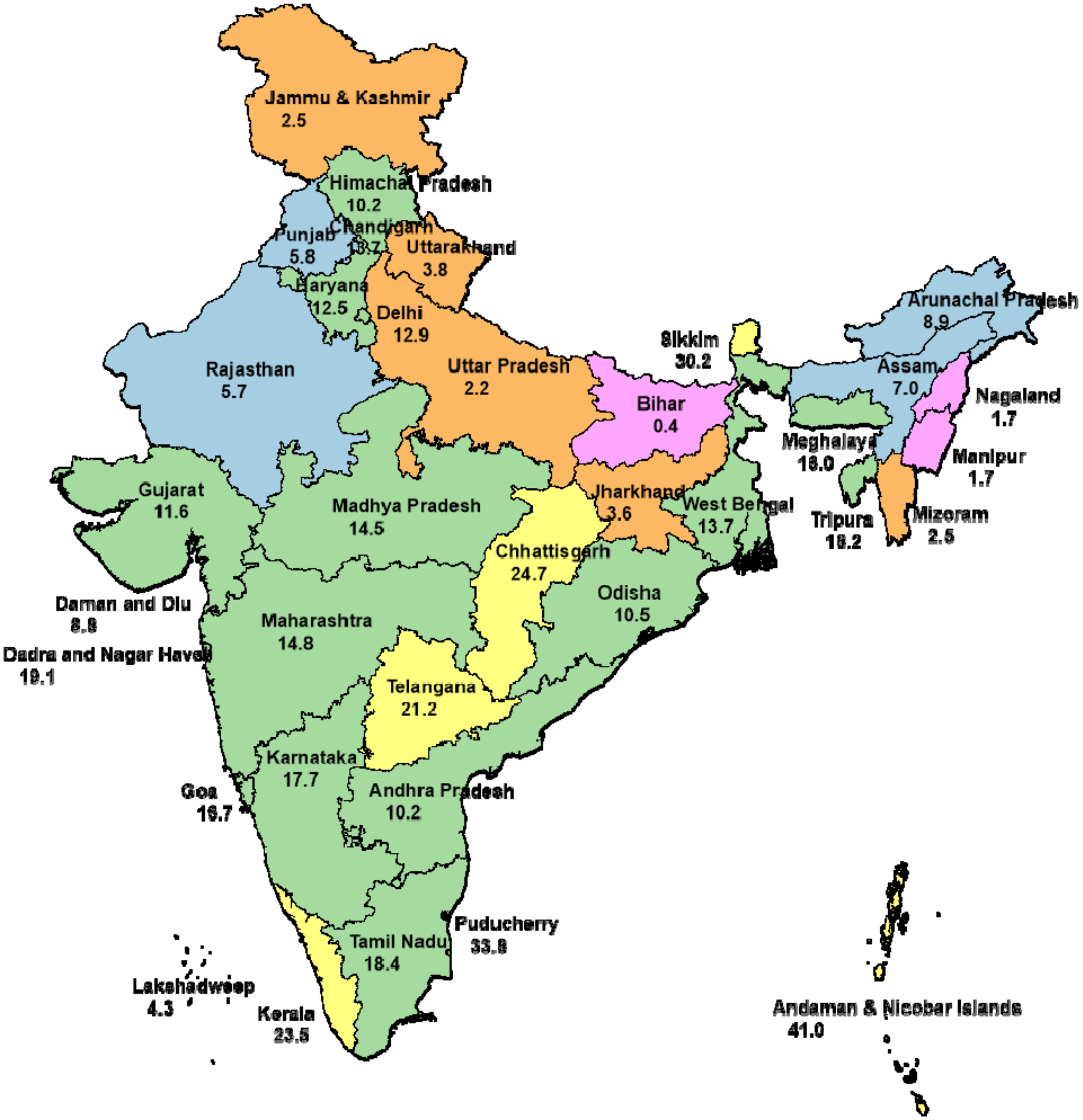

Suicide Rate

All India Average 10.2

- upto 2
- 3 to 5
- 6 to 10
- 11 to 20
- Above 20

Note:
Suicide Rate means Number of Suicides per One Lakh population.

- As per data provided by States/UTs.

Map Powered by DevInfo, UNICEF

Table – 2 (C)
States/UTs with Higher Suicide Rate during 2016 to 2018

Year					
**2016**		**2017**		**2018**	
Sikkim	40.5	Puducherry	40.1	A & N Islands	41.0
Puducherry	33.3	A & N Islands	35.6	Puducherry	33.8
A & N Islands	26.4	Sikkim	32.9	Sikkim	30.2
Chhattisgarh	25.8	Kerala	22.6	Kerala	23.5
Telangana	24.5	Chhattisgarh	22.5	Telangana	21.2
**National Rate**	**(10.3)**	**National Rate**	**(9.9)**	**National Rate**	**(10.2)**

- As per data provided by States/UTs.

Causes of Suicides

'Family Problems' and 'Illness' were the major causes of suicides which accounted for 30.4% and 17.7% of total suicides respectively during 2018. 'Marriage Related Issues' (6.2%), 'Drug Abuse/Addiction' (5.3%) 'Love Affairs' (4.0%), 'Bankruptcy or Indebtedness' (3.7%), 'Failure in Examination' and 'Unemployment' (2.0% each), 'Professional/Career Problem' (1.3%) and 'Property Dispute' (0.9%) were other causes of suicides [**Table–2.4**].

Figure – 2.4
Percentage Share of Various Causes of Suicides During 2018

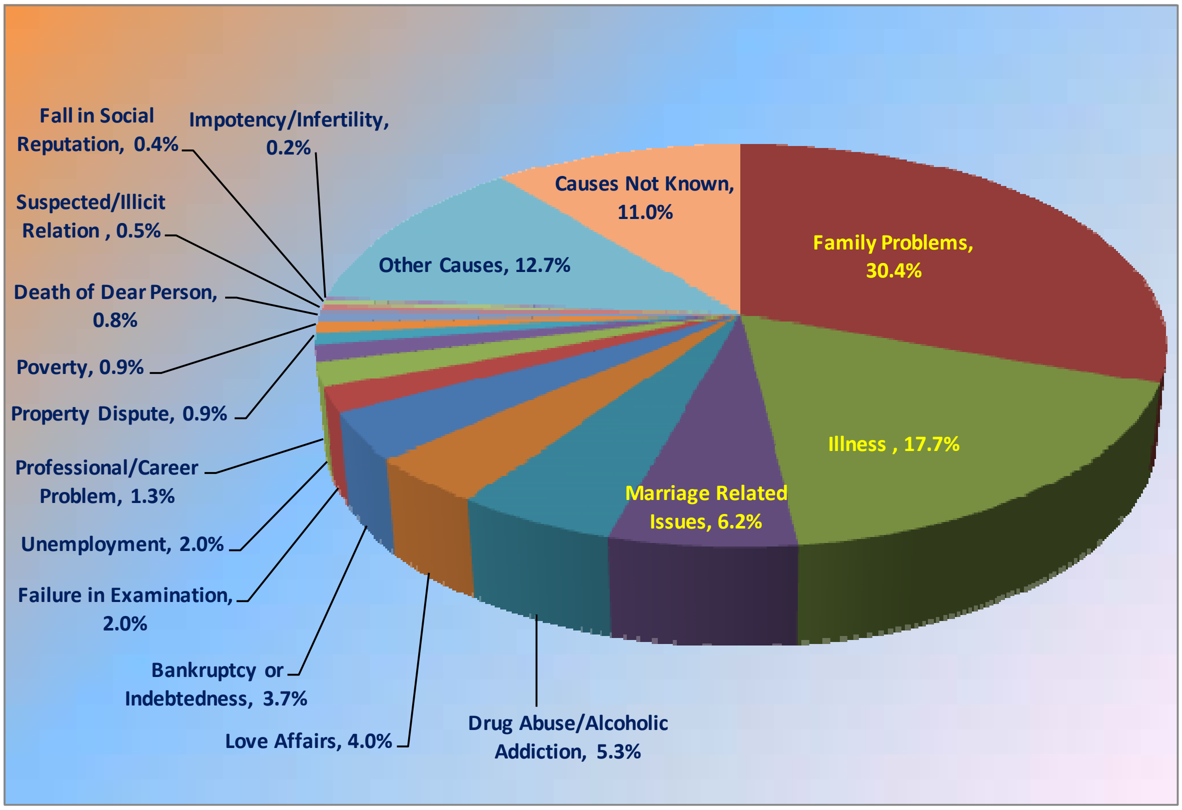

- As per data provided by States/UTs.

* Figure of Suicides due to ideological causes/hero worshiping, physical abuse (rape etc.) and illegitimate pregnancy included along with Other Causes.

State and UT wise and City wise details on various causes of suicides are presented in **Table–2.5**. The States/UTs which reported more than the All-India average share under the two major causes of suicides i.e. 'Illness' and 'Family Problems' are grouped in the **Table–2(D)**.

Suicide Victims by Sex and Age Group

Age group wise distribution of suicides by causes during 2018 is presented in **Table–2(E).** The overall male: female ratio of suicide victims for the year 2018 was 68.5:31.5, which remains same as compared to year 2017 (68.5:31.5). The proportion of female victims were more in 'Marriage Related Issues' specifically in 'Dowry Related Issues', and 'Impotency/Infertility'. The age group (18 and above - below 30 years) and persons above 30 years - below 45 years of age were the most vulnerable groups resorting to suicides. These age groups accounted for 34.9% and 31.6% suicides respectively. 'Family Problems' (2,236), 'Failure in Examination' (1,529), 'Love Affairs' (1,131) and 'Illness' (932) were the main causes of suicides among children (below 18 years of age).

15 States/UTs have reported higher percentage share than the All India average in suicides due to 'Illness'. Similarly, 17 States/UTs have reported higher share than the All India average in suicides committed due to 'Family Problems' during the year 2018.

Professional Status of Suicide Victims

Details on professional status of suicide victims are presented in **Table–2.6**. House-wives accounted for 54.1% of the total female victims (22,937 out of 42,391) and constitute nearly 17.1% of total victims who committed suicides (22,937 out of 1,34,516) during 2018.

Government servants accounted for 1.3% (1,707 out of 1,34,516) of the total suicide victims as compared to 6.1% (8,246 out of 1,34,516) of total victims from Private Sector Enterprises. Employees from Public Sector Undertakings formed 1.5% (2,022 out of 1,34,516) of the total suicide victims, whereas students and un-employed victims accounted for 7.6% (10,159 victims) and 9.6% (12,936 victims) of total suicides respectively. Self-employed category accounted for 9.8% of total suicide victims (13,149 out of 1,34,516).

Table – 2 (D)
States & UTs Reporting Higher Share of Suicides due to Illness and Family Problems during 2018

Illness				Family Problems			
All India % Share							
17.7%				30.4%			
Sl. No.	State/UT	Suicides	% Share	Sl. No.	State/UT	Suicides	% Share
1	Punjab	722	42.1	1	Manipur	32	61.5
2	Sikkim	82	41.2	2	Daman & Diu	21	60.0
3	Lakshadweep	1	33.3	3	Tripura	388	53.9
4	Andhra Pradesh	1610	30.3	4	Chandigarh	84	52.5
5	Puducherry	150	30.0	5	Tamil Nadu	6433	46.3
6	A & N Islands	46	28.0	6	Kerala	3213	39.0
7	Karnataka	3180	27.5	7	Uttar Pradesh	1872	38.6
8	Kerala	2122	25.8	8	Telangana	2972	37.9
9	Goa	60	23.4	9	Uttarakhand	150	35.6
10	Gujarat	1709	21.9	10	Maharashtra	6072	33.8
11	Tamil Nadu	3034	21.8	11	D & N Haveli	34	33.7
12	Haryana	697	19.7	12	Bihar	146	33.0
13	Maharashtra	3530	19.6	13	Odisha	1460	31.8
14	Madhya Pradesh	2279	19.4	14	Jammu & Kashmir	104	31.5
15	Tripura	138	19.2	15	Delhi (UT)	787	31.2
				16	Madhya Pradesh	3649	31.0
				17	Rajasthan	1339	30.9

- As per data provided by States/UTs.

A total of 10,349 persons involved in farming sector (consisting of 5,763 farmers/cultivators and 4,586 agricultural labourers) have committed suicides during 2018, accounting for 7.7% of total suicides victims (1,34,516) in the country. Out of 5,763 farmer/cultivator suicides, a total of 5,457 were male and 306 were female during 2018. Out of 4,586 suicides committed by agricultural labourers during 2018, 4,071 were male and 515 were female.

Certain States/UTs namely, West Bengal, Bihar, Odisha, Uttarakhand, Meghalaya, Goa, Chandigarh, Daman & Diu, Delhi UT, Lakshadweep and Puducherry reported zero suicides of Farmers/Cultivators as well as Agricultural Labourers.

Figure – 2.5

Suicide Victims by Sex and Age Group during 2018

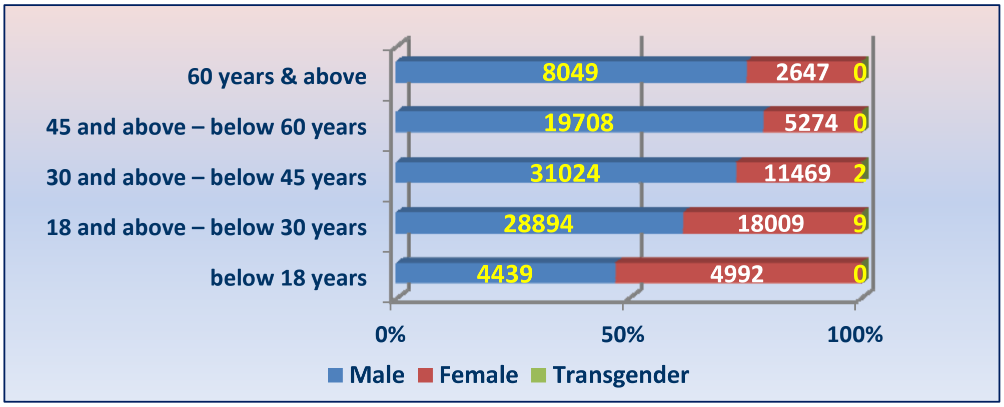

- As per data provided by States/UTs.

Figure – 2.6

Percentage Distribution of Suicide Victims by Profession During 2018

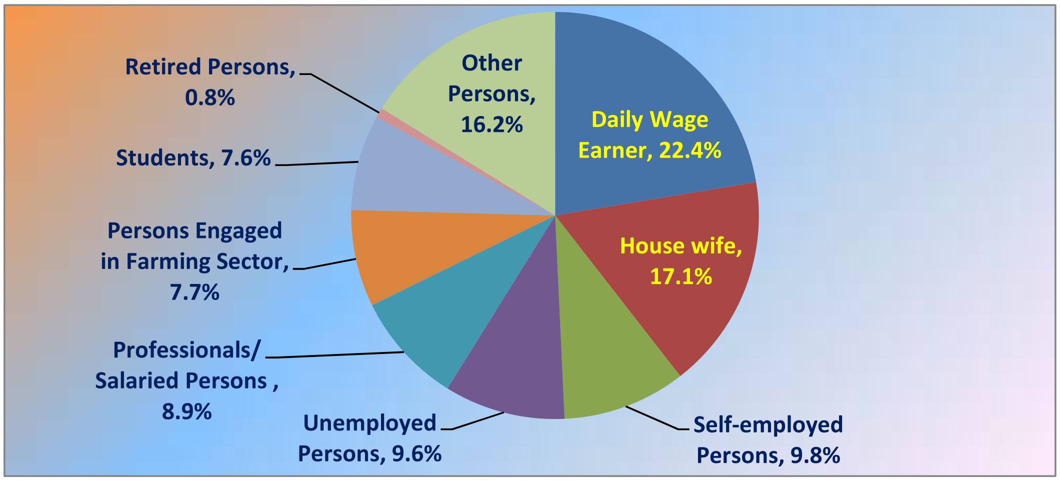

- As per data provided by States/UTs.

Table 2(E)

Age and Gender - wise Distribution of Suicides during 2018
(Cause-wise)

Sl. No.	Cause	Below 18 years				18 yrs. – Below 30 years				30 yrs. – Below 45 years			
		M	F	Tr	T	M	F	Tr	T	M	F	Tr	T
(1)	(2)	(3)	(4)	(5)	(6)	(7)	(8)	(9)	(10)	(11)	(12)	(13)	(14)
1	Bankruptcy or Indebtedness	7	1	0	8	731	123	0	854	1774	179	0	1953
2	**Marriage Related Issues (Total)**	**135**	**163**	**0**	**298**	**1516**	**2926**	**0**	**4442**	**1537**	**1314**	**0**	**2851**
	2.1 Non Settlement of Marriage	30	53	0	83	676	647	0	1323	690	299	0	989
	2.2 Dowry Related Issues	57	23	0	80	51	1343	0	1394	45	455	0	500
	2.3 Extra Marital Affairs	5	10	0	15	272	363	0	635	290	222	0	512
	2.4 Divorce	9	5	0	14	103	147	0	250	143	118	0	261
	2.5 Others	34	72	0	106	414	426	0	840	369	220	0	589
3	Failure in Examination	757	772	0	1529	648	386	0	1034	45	8	0	53
4	Impotency/Infertility	2	7	0	9	35	74	0	109	50	86	0	136
5	Family Problems	979	1257	0	2236	8456	5940	0	14396	9868	4228	1	14097
6	**Illness (Total)**	**416**	**516**	**0**	**932**	**3617**	**2109**	**3**	**5729**	**4817**	**1989**	**0**	**6806**
	6.1 AIDS/STD	2	0	0	2	31	8	0	39	65	11	0	76
	6.2 Cancer	3	6	0	9	128	44	0	172	259	93	0	352
	6.3 Paralysis	11	8	0	19	118	36	0	154	224	65	0	289
	6.4 Insanity/ Mental Illness	247	245	0	492	1969	889	1	2859	2264	858	0	3122
	6.5 Other Prolonged Illness	153	257	0	410	1371	1132	2	2505	2005	962	0	2967
7	Death of Dear Person	16	21	0	37	198	99	0	297	226	119	0	345
8	Drug Abuse/Alcoholic Addiction	46	9	0	55	1745	28	1	1774	2797	64	0	2861
9	Fall in Social Reputation	13	13	0	26	105	51	2	158	144	38	0	182
10	Ideological Causes/Hero Worshipping	7	7	0	14	18	19	0	37	19	10	0	29
11	Love Affairs	466	665	0	1131	2168	1335	0	3503	414	240	0	654
12	Poverty	17	14	0	31	302	59	0	361	398	71	0	469
13	Unemployment	31	9	0	40	1240	180	0	1420	868	95	0	963
14	Property Dispute	24	4	0	28	216	70	0	286	329	66	0	395
15	Suspected/ Illicit Relation (Other than Sl. No. 2.3)	18	13	0	31	166	137	0	303	173	77	0	250
16	Illegitimate Pregnancy (Other than Sl. No. 2.3)	0	5	0	5	0	24	0	24	0	13	0	13
17	Physical Abuse (Rape, etc.)	9	24	0	33	18	23	0	41	29	22	1	52
18	Professional/Career Problem	22	26	0	48	445	121	0	566	566	86	0	652
19	Causes Not Known	639	722	0	1361	3600	2100	1	5701	3208	1250	0	4458
20	Other Causes	835	744	0	1579	3670	2205	2	5877	3762	1514	0	5276
21	**Total**	**4439**	**4992**	**0**	**9431**	**28894**	**18009**	**9**	**46912**	**31024**	**11469**	**2**	**42495**

Note: 'M'- refers to Male, 'F'- refers to Female, 'Tr'- refers to Transgender and 'T' – Total

- **As per data provided by States/UTs.**

Table 2(E)

Age and Gender - wise distribution of Suicides during 2018 (Concluded) (Cause-wise)

Sl. No.	Cause	45 yrs. – Below 60 years				60 years & above				Total			
		M	F	Tr	T	M	F	Tr	T	M	F	Tr	T
(1)	(2)	(15)	(16)	(17)	(18)	(19)	(20)	(21)	(22)	(23)	(24)	(25)	(26)
1	Bankruptcy or Indebtedness	1562	127	0	1689	434	32	0	466	4508	462	0	4970
2	**Marriage Related Issues (Total)**	**395**	**180**	**0**	**575**	**107**	**11**	**0**	**118**	**3690**	**4594**	**0**	**8284**
	2.1 Non Settlement of Marriage	125	39	0	164	24	2	0	26	1545	1040	0	2585
	2.2 Dowry Related Issues	10	30	0	40	1	1	0	2	164	1852	0	2016
	2.3 Extra Marital Affairs	66	34	0	100	0	1	0	1	633	630	0	1263
	2.4 Divorce	43	32	0	75	45	1	0	46	343	303	0	646
	2.5 Others	151	45	0	196	37	6	0	43	1005	769	0	1774
3	Failure in Examination	2	5	0	7	1	1	0	2	1453	1172	0	2625
4	Impotency/Infertility	24	15	0	39	4	0	0	4	115	182	0	297
5	Family Problems	5614	1873	0	7487	2058	661	0	2719	26975	13959	1	40935
6	**Illness (Total)**	**4483**	**1552**	**0**	**6035**	**2941**	**1321**	**0**	**4262**	**16274**	**7487**	**3**	**23764**
	6.1 AIDS/STD	39	7	0	46	9	0	0	9	146	26	0	172
	6.2 Cancer	339	108	0	447	199	88	0	287	928	339	0	1267
	6.3 Paralysis	332	69	0	401	183	75	0	258	868	253	0	1121
	6.4 Insanity/ Mental Illness	1654	609	0	2263	967	431	0	1398	7101	3032	1	10134
	6.5 Other Prolonged Illness	2119	759	0	2878	1583	727	0	2310	7231	3837	2	11070
7	Death of Dear Person	155	69	0	224	119	51	0	170	714	359	0	1073
8	Drug Abuse/Alcoholic Addiction	1863	45	0	1908	588	7	0	595	7039	153	1	7193
9	Fall in Social Reputation	114	18	0	132	23	3	0	26	399	123	2	524
10	Ideological Causes/ Hero Worshipping	12	0	0	12	2	0	0	2	58	36	0	94
11	Love Affairs	27	20	0	47	2	5	0	7	3077	2265	0	5342
12	Poverty	236	26	0	262	70	9	0	79	1023	179	0	1202
13	Unemployment	237	21	0	258	55	5	0	60	2431	310	0	2741
14	Property Dispute	342	66	0	408	77	15	0	92	988	221	0	1209
15	Suspected/ Illicit Relation (Other than Sl. No. 2.3)	49	16	0	65	4	0	0	4	410	243	0	653
16	Illegitimate Pregnancy (Other than Sl. No. 2.3)	0	2	0	2	0	0	0	0	0	44	0	44
17	Physical Abuse (Rape, etc.)	40	14	0	54	5	7	0	12	101	90	1	192
18	Professional/Career Problem	293	43	0	336	83	12	0	95	1409	288	0	1697
19	Causes Not Known	2008	487	0	2495	599	214	0	813	10054	4773	1	14828
20	Other Causes	2252	695	0	2947	877	293	0	1170	11396	5451	2	16849
21	**Total**	**19708**	**5274**	**0**	**24982**	**8049**	**2647**	**0**	**10696**	**92114**	**42391**	**11**	**134516**

Note: 'M'- refers to Male, 'F'- refers to Female, 'Tr'- refers to Transgender and 'T' – Total

- **As per data provided by States/UTs.**

Out of total 92,114 male suicides, maximum suicides were committed by daily wage earners (26,589) followed by self-employed persons (12,175) and Unemployed Persons (10,687). A total of 42,391 females committed suicides during 2018 in the country. 22,937 females who committed suicides were house-wives followed by students (4,790) and daily wage earners (3,535). A total of 11 transgender have committed suicide. Out of 11 transgender, 3 were daily wage earners, 1 each were 'Professionals/Salaried Persons' and 'Unemployed Persons' while 6 falls under 'Other Persons'.

State/UT-wise and profession wise analysis of suicide victims is presented in **Table–2.7**. Majority of suicides committed by house-wives were reported in Madhya Pradesh (2,876 out of 22,937 suicides) followed by Maharashtra (2,743 suicides) and Tamil Nadu (2,472 suicides) which accounted for 12.5%, 12.0% and 10.8% of total such suicides during 2018 respectively. Majority of suicides committed by government servants were reported in Maharashtra (231 out of 1,707 suicides) followed by Tamil Nadu (173 suicides), Madhya Pradesh (155 suicides), Karnataka (137 suicides) and Haryana (124 suicides) which accounted for 13.5%, 10.1%, 9.1%, 8.0% and 7.3% of total such suicides respectively. Majority of suicides committed by persons engaged in Private Sector Enterprises were reported in Maharashtra (1,749 out of 8,246 suicides), Tamil Nadu (1,146 suicides), West Bengal (778 suicides), Karnataka (760 suicides) and Gujarat (685 suicides), they accounted for 21.2%, 13.9%, 9.4%, 9.2% and 8.3% of total such suicides respectively. 14.3% of total suicides committed by students were reported in Maharashtra (1,448 suicides) followed by 9.4% in Tamil Nadu (953 suicides), 8.5% in Madhya Pradesh (862 suicides), 7.4% in Karnataka (755 suicides) and 6.0% in West Bengal (609 suicides). Majority of suicides committed by un-employed persons, 12.3% were in Kerala (1,585 out of 12,936 suicides), 12.2% in Tamil Nadu (1,579 suicides), 9.7% in Maharashtra (1,260 suicides), 8.5% in Karnataka (1,094 suicides) and 7.0% in Uttar Pradesh (902 suicides). Majority of suicides committed by persons engaged in business activities were reported by Karnataka (13.9%), Maharashtra (12.1%), Tamil Nadu (11.7%), West Bengal (8.9%) and Kerala (8.1%). Majority of victims engaged in farming sector were reported in Maharashtra (34.7%), Karnataka (23.2%), Telangana (8.8%), Andhra Pradesh (6.4%) and Madhya Pradesh (6.3%).

Social Status of Suicide Victims

Social Status of victims is classified in seven categories namely 'Un-married', 'Married', 'Widowed/Widower', 'Divorcee', 'Separated', 'Others' and 'Status Not Known'.

The information on the social status of suicide victims is presented in **Table– 2.8(I)**. It is observed that 68.8% (92,533 out of 1,34,516) of the suicide victims were married while 22.7% were un-married (30,503). Widowed/Widower, Divorcees and Separated have accounted for 1.7% (2,329 victims), 0.7% (943 victims) and 0.6% (849 victims) of total suicide victims respectively during 2018.

The State/UT-wise information on the social status of suicide victims is presented in **Table–2.9**.

Economic Status of Suicide Victims

The information on the economic status of suicide victims is presented in **Table– 2.8(II)**. 66.2% of suicide victims in 2018 were having annual income of less than ₹1 lakh (88,986 out of 1,34,516 victims). 29.1% (39080 out of 1,34,516 victims) of suicide victims belong to annual income group of '₹1 lakh & above to less than ₹5 lakh'. The State/UT wise information on the Economic Status of suicide victims is presented in **Table–2.10**.

Educational Status of Suicide Victims

The sex-wise and education-wise break-up of suicide victims is presented in **Table-2.8(III)**. The maximum numbers of suicide victims (23.6%) (31,757 out of 1,34,516 victims) were educated up to Matriculation/ Secondary level. Middle level educated, Primary educated, Hr. Secondary/ Intermediate/Pre-University educated and Illiterate persons accounted for 19.5% (26,252 out of 1,34,516 victims), 17.1% (23,048 out of 1,34,516 victims), 16.4% (22,076 out of 1,34,516 victims) and 12.7% (17,026 out of 1,34,516 victims) respectively of total suicide victims during 2018. Only 3.3% (4,437 out of 1,34,516 victims) of total suicide victims were graduates and above during 2018.

Figure – 2.7
Distribution of Suicide Victims by Social Status during 2018

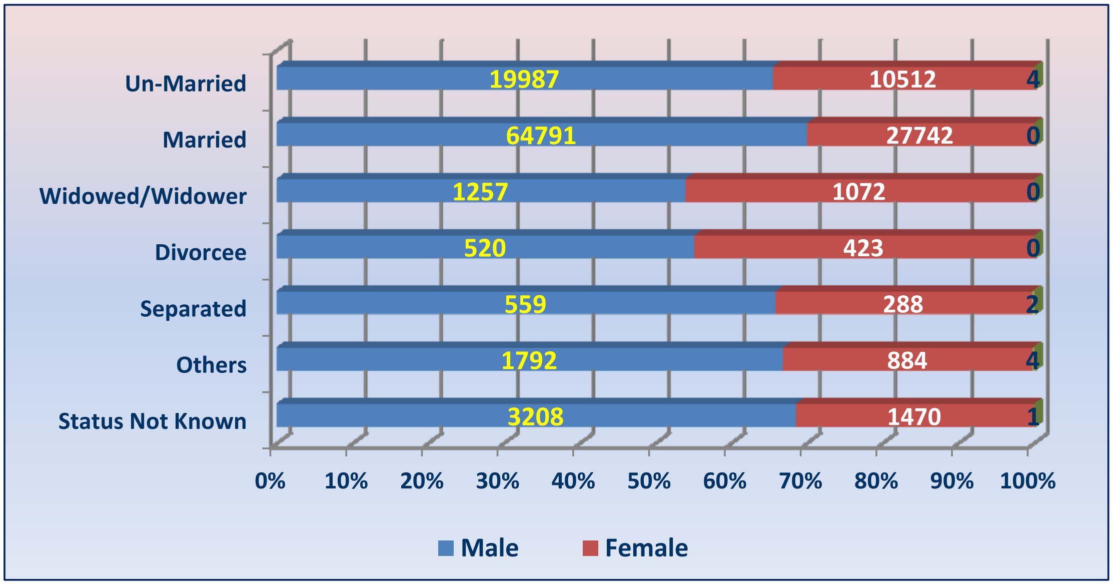

- As per data provided by States/UTs.

Figure – 2.8
Percentage Share of Suicide Victims by Educational Status during 2018

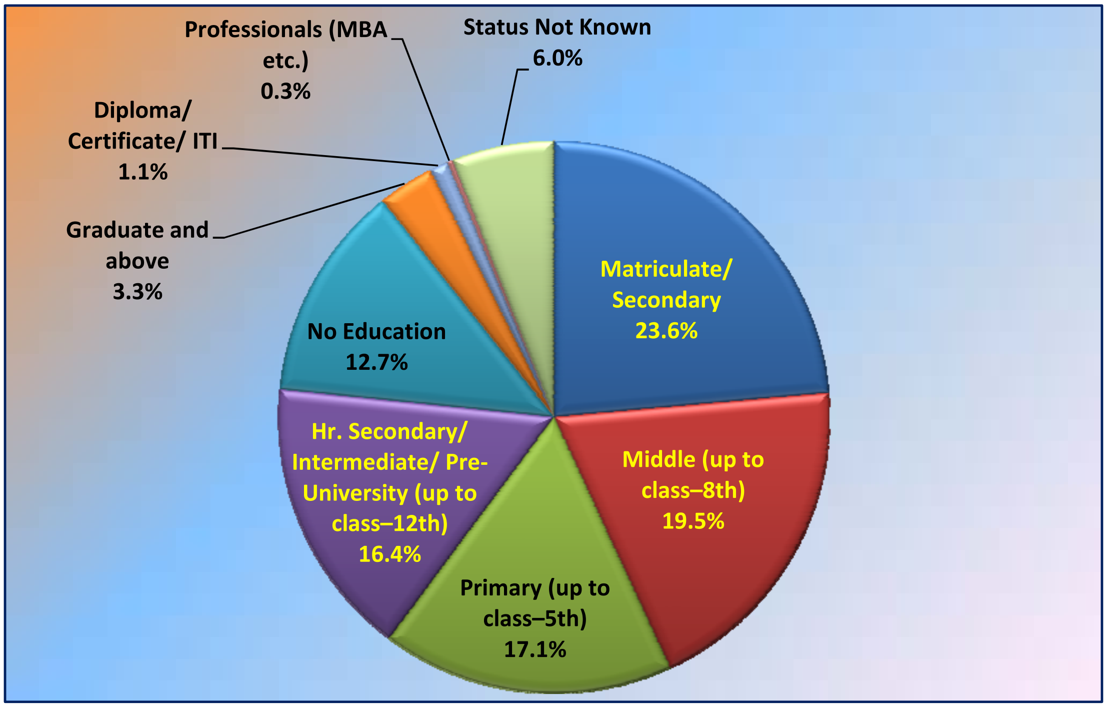

- As per data provided by States/UTs.

Table – 2 (F)
Percentage of Suicide Victims by Educational Level during 2016 – 2018

Sl. No.	Educational Level	Percentage Share		
		2016	2017	2018
(1)	(2)	(3)	(4)	(5)
1	No Education	13.8	13.0	12.7
2	Primary Level	18.3	16.3	17.1
3	Middle Level	20.1	19.4	19.5
4	Matriculate/Secondary Level	22.6	23.6	23.6
5	Higher Secondary Level	12.8	15.9	16.4
6	Diploma	1.1	1.2	1.1
7	Graduate & above	3.1	3.5	3.3
8	Professionals (MBA etc.)	0.2	0.3	0.3
9	Status Not Known	8.1	6.8	6.0
**10**	**Total**	**100.0**	**100.0**	**100.0**

- As per data provided by States/UTs.

The State/UT wise information on the Education Status of suicide victims is presented in **Table– 2.11**.

Out of 31,757 suicides committed by persons educated up-to matriculate/secondary level, 17.2% were reported in Maharashtra followed by Kerala (10.4%) and Tamil Nadu (10.1%). Out of 26,252 suicides committed by persons educated up-to middle level, 14.5% were reported in Maharashtra followed by Tamil Nadu (11.6%) and Madhya Pradesh (10.0%). Out of 17,026 suicides committed by persons with no education 15.5% were reported in Telangana followed by Madhya Pradesh (12.3%) and Tamil Nadu (11.1%) **[Table–2.11]**.

Percentage share of suicide victims by educational level during 2016-18 is presented in **Table–2(F)**.

Means Adopted for Committing Suicides

The means adopted for committing suicide varied from the easily available and effective means such as consumption of poison, jumping etc. to more painful means such as self-inflicted injuries, hanging etc. Like previous year, 'Hanging' (51.5%), consuming 'Poison' (26.7%), 'Drowning' (4.9%) and 'Fire/Self-Immolation' (4.4%) were the prominent means/mode of committing suicide. **[Table – 2.12]**

Percentage share of the means adopted in committing suicide during 2017-18 is presented in **Table–2(G)**.

During the year 2018, the share of 'Fire/Self Immolation (from 5.3% in 2017 to 4.4% in 2018) and 'By Poison' (from 27.5% in 2017 to 26.7% in 2018) as mode adopted by suicide victims has decreased while share of 'Hanging' (from 49.8% in 2017 to 51.5% in 2018), 'By Drowning (from 4.8% in 2017 to 4.9% in 2018), and 'By Jumping' (from 1.7% in 2017 to 1.9% in 2018) have increased during 2018 over 2017. **[Table – 2(G)]**

The sex-wise break-up and means adopted for suicide in various States/UTs is presented in **Table–2.13**.

Table – 2 (G)
Percentage of Means/Mode Adopted by Victims to Commit Suicide during 2017-2018

SL.	Means/Mode Adopted	Percentage & Number	
		2017	2017
(1)	(2)	(3)	(4)
1	Consuming Sleeping Pills	0.8% (1,015)	0.7% (939)
2	Drowning	4.8% (6,235)	4.9% (6,579)
3	Fire/Self Immolation	5.3% (6,858)	4.4% (5,950)
4	Firearms	0.4% (480)	0.4% (521)
5	By Hanging	49.8% (64,705)	51.5% (69,306)
6	By Poison	27.5% (35,671)	26.7% (35,862)
7	By Self inflicting Injury	0.8% (975)	0.6% (772)
8	By Jumping	1.7% (2,203)	1.9% (2,557)
9	By Coming under Running Vehicles/Trains	2.9% (3,816)	2.9% (3,848)
10	By Touching Electric Wire	0.4% (560)	0.4% (565)
11	By Other Means	5.7% (7,369)	5.7% (7,617)
**12**	**Total**	**100.0**	**100.0**

Note: Bracket ( ) refer to Number of victims who have committed suicides

- As per data provided by States/UTs.

The number of male victims were more than females in all means of suicide except those who committed suicides by 'Fire/Self-immolation' where share of female victims was more (3,809 out of 5,950).

Suicides in Cities

City refers to mega city i.e. city having population of 10,00,000 or more.

The numbers of suicides in 53 mega cities show an increasing trend during 2015 to 2018. It increased by 6.2% (from 19,665 in 2015 to 20,879 in 2016) and increased by 1.8% (from 20,879 in 2016 to 21,240 in 2017). It increased by 0.8% (from 21,240 in 2017 to 21,408 in 2018). Details on year-wise incidents in cities, percentage share to All-India, rate of suicides and percentage change during 2015 - 2018 may be seen in **Table–2(H)**.

Table – 2 (H)

Incidence of Suicides in Mega-Cities, percentage share to All-India, rate of suicides and percentage change over previous year during 2015 - 2018

Year	Suicides in Cities	Cities Share to All India	Rate in Cities	% Variation over Previous Year (Incidence)
2015	19,665	14.7	12.2	0.3
2016	20,879	15.9	13.0	6.2
2017	21,240	16.4	13.2	1.8
2018	21,408	15.9	13.3	0.8

- As per data provided by States/UTs.

The four Metropolitan Cities — Chennai (2,102), Delhi City (2,369), Bengaluru (2,082) and Mumbai (1,174) have reported higher number of suicides. These four cities together have reported almost 36.1% of the total suicides reported from 53 mega cities. Kolkata and Chennai Cities have shown a declining trend during 2018 over 2017. Delhi City has shown an increase of 8.2% (from 2,189 suicides in 2017 to 2,369 suicides in 2018) and Bengaluru has observed an increase of 2.5% (from 2,032 suicides in 2017 to 2,082 suicides in 2018). [**Table - 2.3**].

The suicide rate in cities (13.3) was higher as compared to All-India suicide rate (10.2). Kollam followed by Durg-Bhilainagar have reported the highest suicides rate of 35.4 and 33.1 respectively. [**Table-2.2**].

'Family Problems (other than marriage related issues)' was the major cause of suicide in cities which accounted for 37.7% (8,069 out of 21,408) of total suicides followed by 'Illness' (17.7%) (3,788 victims out of 21,408 victims). However, a total of 1,027 victims have committed suicide in 53 mega cities due to 'Marriage Related Issues' accounting for 4.8% of total suicides in the cities.

Mass/Family Suicides

This section provides information on the number of cases where family members have committed suicide jointly. Out of 36 States/UTs, Mass/Family Suicides were reported in 11 States during the year 2018. State/UT & City-wise information on mass/family suicides is presented in **Table–2.14.**

A total of 64 cases of mass/family suicides were registered during the year 2018. In these 64 cases of mass/family suicides, a total of 173 persons, comprising of 119 married persons and 54 unmarried persons, have committed suicides during 2018.

Maximum cases of mass/family suicides were reported Tamil Nadu (15 cases) followed by Andhra Pradesh (11 cases), Madhya Pradesh (9), Punjab & Rajasthan (6 cases each) and Kerala (5 cases) during 2017. A total 35 persons in Tamil Nadu, 26 persons in Andhra Pradesh, 24 persons in Kerala and 22 persons in Uttarakhand have committed mass/family suicides during 2018 [**Table-2.14**].

Out of 53 cities, mass/family suicides were reported in 6 cities. These six cities have registered 9 cases of mass/family suicides in which 22 persons have committed suicides during 2018. Among these 22 persons, 17 were married and 5 were unmarried persons [**Table-2.14**].

****
